# Compounding hazards increase flood economic losses across Europe

**DOI:** 10.1038/s41467-026-73248-0

**Published:** 2026-05-19

**Authors:** Michele Ronco, Aloïs Tilloy, Christina Corbane, Damien Delforge, Luc Feyen, Wiebke S. Jäger, Alessia Matanó, Dominik Paprotny, Andrea Sibilia, Timothy Tiggeloven, Philip J. Ward

**Affiliations:** 1 European Commission, Joint Research Centre, Ispra, Italy; 2 Institute of Health and Society (IRSS), University of Louvain (UCLouvain), Brussels, Belgium; 3 Institute for Environmental Studies, Vrije Universiteit Amsterdam, Amsterdam, The Netherlands; 4 Research Department Transformation Pathways, Potsdam Institute for Climate Impact Research (PIK), Potsdam, Germany; 5 Institute of Marine and Environmental Sciences, University of Szczecin, Szczecin, Poland; 6 Baltic Climate Centre, University of Szczecin, Szczecin, Poland; 7 Unisystems Luxembourg S.a.r.l. External Service Provider of European Commission Joint Research Centre, Ispra, Italy; 8 CMCC Foundation, Euro-Mediterranean Center on Climate Change, Venice, Italy, Venice, Italy; 9 Deltares, Delft, The Netherlands

**Keywords:** Natural hazards, Governance, Water resources

## Abstract

Compound events-combinations of multiple hazards contributing to societal or environmental risk-can significantly exacerbate disaster impacts, yet their effect on flood-related losses remains poorly quantified. Using a pan-European multi-hazard dataset spanning 1981-2020 at sub-national resolution, we find that more than 70% of recorded flood events involve compounding hazards, including meteorological extremes such as heatwaves and windstorms, alongside anomalous river discharge, with an increasing trend over time. The top 1% of events by economic losses are all compound, with total losses exceeding 167 billion euros above single-hazard floods. We introduce a compound hazard complexity metric and combine it with regional exposure and vulnerability data. Applying an ensemble machine learning model with explainable AI and a Double Machine Learning Causal Forest, we show that regions with higher complexity experience greater losses, even after controlling for flood magnitude and vulnerability, highlighting the importance of compound hazard information in risk modeling.

## Abstract (web summary)

Over 70% of European floods involve compounding hazards such as heatwave and windstorm, with compound events causing 167 billion euros more in losses. AI methods reveal that regions with a higher mix of compound hazards experience greater losses.

## Introduction

Disasters rarely result from a single hazard acting in isolation. Instead, they frequently stem from the interplay of multiple hazards—such as extreme wind, river flooding, or drought—that combine to amplify hazardous conditions and shape complex impact pathways^1^. These compound events—combinations of multiple hazards that contribute to risk^2^—can substantially exacerbate disaster outcomes, often producing consequences more severe than those caused by any individual hazard alone^3,4^. Yet, their role in shaping economic, societal, and ecological impacts remains not fully understood, largely due to limitations in data availability, methodologies, and operational frameworks^5^. Consequently, disaster risk assessments often overlook the compound nature of these events, increasing the likelihood of underestimated risks. This can lead to inadequate preparedness, suboptimal response strategies, and missed opportunities for risk reduction. While recent research advocates for a shift toward integrated, “compound-aware” approaches^6^, putting these ideas into practice remains challenging. In particular, there is a pressing need for large-scale, impact-linked evidence to better understand how compound events influence disaster outcomes across diverse contexts. In this study, we address this gap by examining how compound hazards shape flood impacts, which are among the most systematically recorded and economically relevant disasters in Europe^7–13^.

A primary hazard event may amplify subsequent hazards or heighten social vulnerability, leading to increased impact. Illustrating the dynamics of compound hazards, Emilia-Romagna experienced severe flooding in May 2023 after a series of intense rainfall events occurred following a two-year drought^14^. This drought-induced soil cracking reduced infiltration capacity, hindering the catchment’s ability to absorb the deluge^15,16^. Dynamics associated with the sequential occurrence (preconditioning, temporally compounding) or co-occurrence (multivariate) of natural hazards are conceptually well established^17–19^. It remains unclear whether compound events consistently lead to greater impacts across regions, timescales, and event types, as a result of limited empirical evidence. Most insights stem from isolated case studies or post-disaster assessments-often qualitative and lacking statistical generalizability-with a few notable exceptions that have begun to systematically explore these dynamics^20–24^. This gap has historically reflected a limited quantitative understanding of hazard interrelations and the unavailability, inconsistency, and unreliability of risk-related data (exposure, vulnerability, impact). However, recent advances in multi-hazard analysis, coupled with improvements in disaster data collection and management, are beginning to address these limitations—unlocking new opportunities to systematically investigate compound processes and their impacts at scale^25–28^.

Our analysis starts from the HANZE database^29,30^, which harmonizes flood impacts from multiple sources, and enhances it by integrating hydrometeorological hazard information to produce a pan-European flood-related multi-hazard dataset spanning 1981–2020 at subnational resolution. We focus on five compound hazard types, described in Table 1: wet sequences, drought-flood, heatwave-flood (hot–wet sequences), coldwave-flood (compound cold events), and windstorm-flood events. Wet sequences represent floods following antecedent high-flow conditions, reflecting pre-wetted catchments that amplify flood magnitude^31–33^. Drought-flood events occur when heavy precipitation follow prolonged dry periods, where compacted soils increase runoff^34–36^. Hot–wet sequences capture floods shortly after heatwaves, often linked to thunderstorm formation under extreme temperatures^37–39^. Coldwave-flood events arise when floods coincide with or follow cold spells, including snowmelt or ice-jam-driven floods^40,41^. Windstorm-flood events involve floods coinciding with extreme wind, typically from extratropical cyclones, combining direct and indirect impacts^42–44^.

**Table 1:** Overview of the five climate-related compound hazard types examined in this study-flood sequences, drought to flood transitions, hot–wet sequences, coldwave flood events, and wind-flood compounding-showing their temporality relative to flood occurrences, as well as possible interrelations with flood hazard and vulnerability

Compound events	Definition	Hazard interrelation type	Temporality	Hazard interrelation	Vulnerability interrelations
Wet sequence	Period of wet conditions preceding a flood impact event.	Precondition: temporally compound	Before: [−56, −4] days	Antecedent wet conditions increase flood intensity	Damaged or weakened infrastructures from previous floods
Drought-flood	Flood impact event occurs during or soon after a drought.	Multivariate: Temporally compound	Before, during: [−56, 0] days	Storage depletion reduces flood peak, Dry soil increases surface runoff	More vulnerable society - negative impact of drought mitigation measures - impact on cropland
Cold-flood	Flood event occurs during or rapidly after a cold wave.	Precondition	Before, during: [−14, 0] days	Higher runoff on frozen grounds, Ice jams in rivers, snowmelt, rain-on-snow events	Hypothermia - excess mortality - mobility disruption - impact on labor productivity
Heat-flood	Flood impact event occurs rapidly after a heatwave.	Precondition	Before, during: [−14, 0] days	Heat fuels intense thunderstorms	More vulnerable society - excess mortality - mobility disruption - impact on labor productivity
Windstorm-flood	Extreme wind precedes or co-occurs with a flood impact event.	Multivariate: temporally compound	Before, during: [−56, 0] days	Same weather system brings heavy precipitation and strong winds; weather systems can occur in sequence	Damages from wind - hampered response

First, we treat each HANZE flood as an individual record and combine it with compound hazard information to generate an event-level compound dataset. Following the typology of Zscheischler et al.^2^, we consider the compound hazards described above that occur within the same NUTS3 region over the temporal windows specified in Table 1, including preconditioning, temporally compound, and multivariate events. We examine and compare yearly temporal trends in single versus compound events across Europe using the Mann–Kendall test, and test whether compound events are associated with systematically higher recorded economic losses than single flood events using permutation tests.

Second, we aggregate these records at the NUTS3 scale, linking all flood events with local exposure, vulnerability, and compound hazard occurrences. We then compute the compound hazard complexity index that summarizes the mix of sequential and concurrent hazard types across all events within each region. This index, together with aggregated exposure, vulnerability, and average flood magnitude, is used in an ensemble machine-learning framework to predict mean log-transformed flood losses. We complement this with a causal analysis using Double Machine Learning and a Causal Forest decomposition^45^ to test whether hazard complexity is associated with increased losses after controlling for confounders. Together, these complementary approaches provide robust evidence that regions experiencing more complex compound hazards tend to incur higher average flood losses, highlighting the importance of considering compound hazards in risk assessment and preparedness strategies.

## Results

### Revisiting historical flood records through a compound hazard lens

**Fig. 1:**
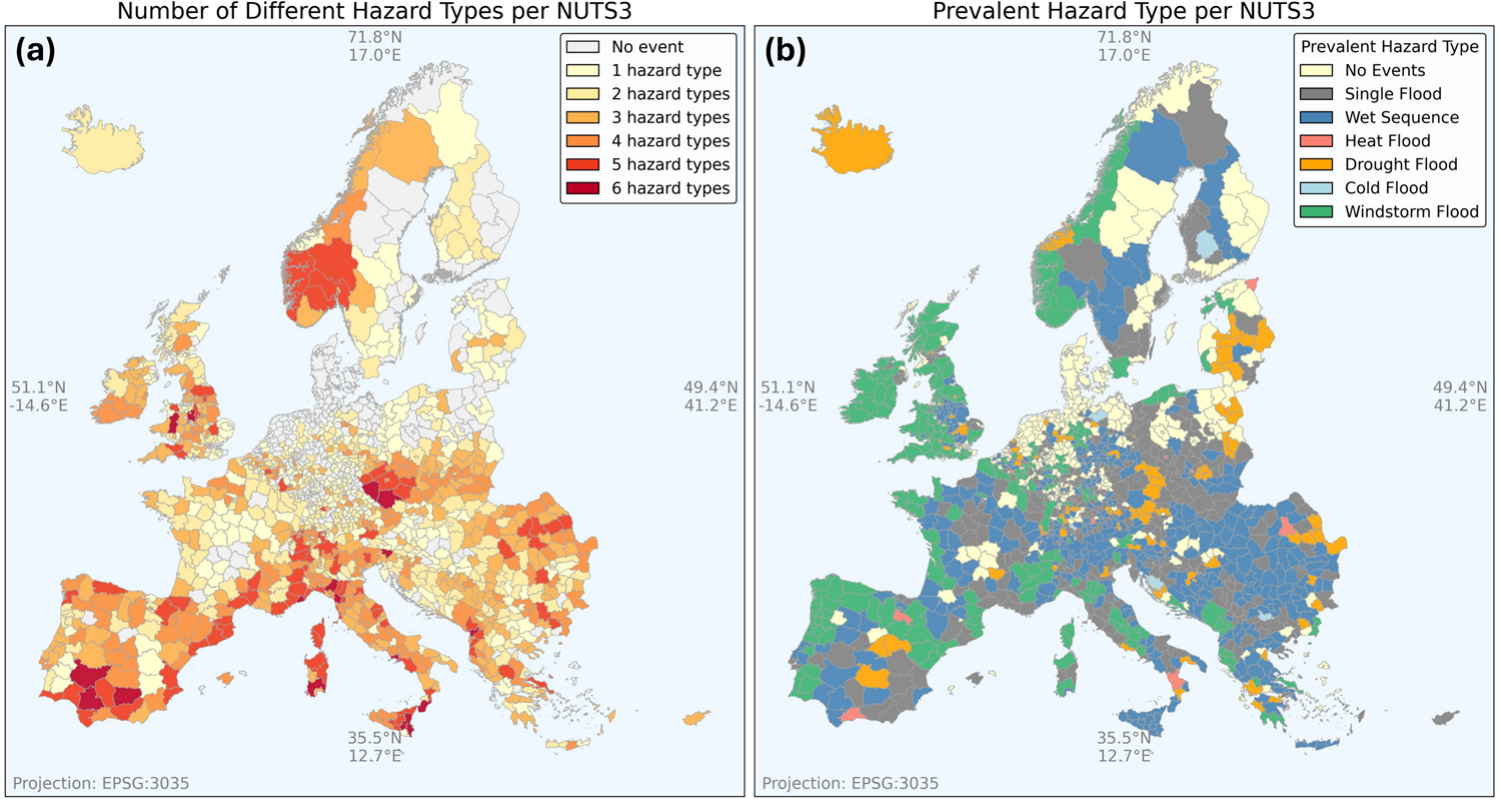
Compound event patterns across Europe at the NUTS-3 level. **a** Number of distinct hazard types recorded per region (0–6) and **b** most frequent hazard type from 1981 to 2020.

We investigate the role of five compound hazard types in shaping flood economic losses across Europe by leveraging the HANZE database^29,30^, following the criteria specified in Table 1 (see also “Methods” and Supplementary Information). Across Europe, we identify a total of 1349 distinct flood events. These occurred within the 1443 NUTS3 regions of Europe, of which 75% experienced at least one flood during 1981–2020 (Fig. 1). On average, each event impacts nearly three NUTS3 regions, resulting in 4306 event-region observations. Based on the criteria in Table 1, we find that 390 events are classified as single-flood events, whereas 959 events satisfy at least one of the five compound-hazard conditions (see also Supplementary Tables 1,  2, and  3).

Spatial patterns reveal that Southern, Central, and Mediterranean Europe exhibit the highest diversity of hazard types, with many NUTS3 regions experiencing three to six distinct hazard combinations (Fig. 1a). In contrast, Northern and Western Europe are dominated by regions with either no recorded floods or only one hazard type. Single-flood events are most common in parts of Central and Eastern Europe, whereas windstorm-flood events prevail across Mediterranean and Atlantic areas, and wet-sequence floods dominate in large parts of Central and Southeastern Europe (Fig. 1b). Drought-flood combinations, though less frequent, appear in specific climatic contexts such as the Baltic region, consistent with prior findings^46^.

**Fig. 2:**
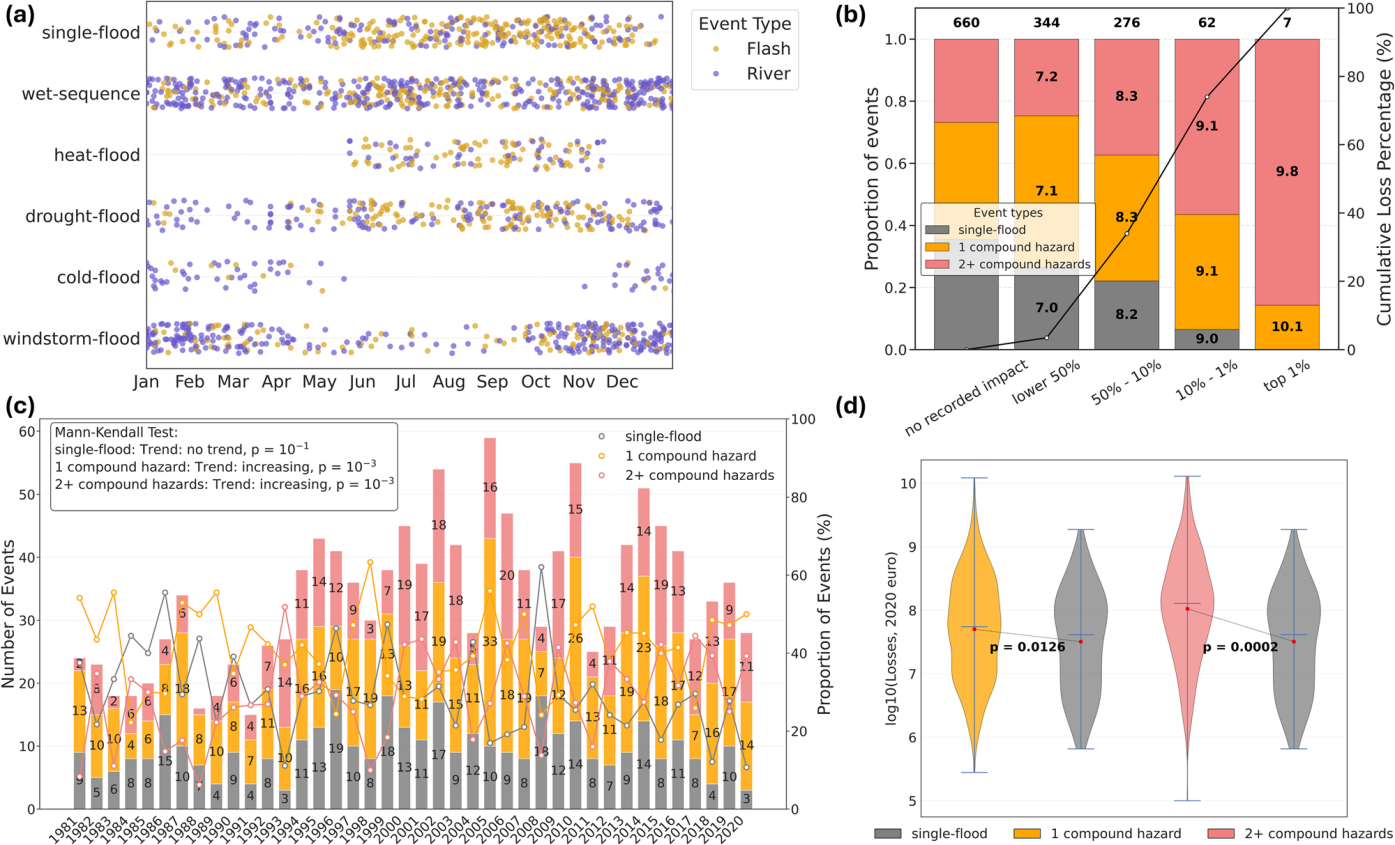
Revisiting flood impacts through a compound hazard lens. **a** Seasonal distribution of flood events by hazard pair category, colored by flood type (flash or riverine); overlapping hazard pairs are shown where a single flood contributes to multiple categories. **b** Share of three mutually exclusive event types across flood-impact classes: “single-flood” (gray), “1 compound hazard” (orange), and “2 + compound hazards” (red). Each bar segment reports the mean log loss for that event type. The impact classes range from no recorded loss, to the lower 50% of losses, 50–10%, 10–1%, and then events with losses in the top 1%, all with respect to the distribution of all observed flood losses. The total number of events per impact class is shown above each bar. The black line overlaid on the stacked bars indicates the cumulative loss distribution. **c** Temporal evolution of the three event types from 1981 to 2020, with lines showing the Mann–Kendall trend for “single-flood” (gray), “1 compound hazard” (orange), and “2 + compound hazards” (red) events. **d** Distribution of log-transformed flood losses across the three event types, shown as violin plots; annotated *P* values correspond to permutation tests comparing the loss distributions.

Seasonal distributions of compound hazard types (Fig. 2a) reveal that windstorm-floods are mostly associated with wintertime riverine floods occurring from October to April, drought-floods from June to December, and single-hazard floods from May to November, whereas wet sequences occur year-round. Finally, windstorm-flood, wet sequences, and cold-flood are predominantly linked to riverine floods, while heat-flood, drought-flood, and single-flood events are more associated with flash floods. These spatiotemporal patterns align with previous characterizations of compound events^31,36,42,43^. While all main results rely on the physically informed temporal window defined in Table 1, we additionally assessed the robustness of event classifications using short (14-day), medium (28-day), and long (56-day) overlap windows, with corresponding distributions provided in Supplementary Fig. 1b (see Supplementary Information file).

We categorize events into three mutually exclusive classes: “single-flood”, “1 compound hazard”, and “2 + compound hazards”. For instance, a flood meeting both drought-flood and heat-flood criteria is classified as a “2 + compound hazards” event. Figure 2c illustrates the annual frequency of these events across Europe from 1981 to 2020 and the corresponding annual proportion for each category. Our analysis reveals distinct trends in compound hazard events over the study period. Decadal averages indicate that “1 compound hazard” floods increased from ~9.7 per year in the 1980s to 16.7 per year in the 2010s, marking a 73% increase (67% when considering 5-year averages). Similarly, “2 + compound hazards” floods surged from 4.3 to 12.4 events per year, a 186% increase (149% for 5-year averages). In contrast, “single-flood” events showed a slight increase from around 8 per year in the 1980s to 9.3 per year in the 2010s, representing a 16% increase (15% decrease for 5-year averages). Mann–Kendall tests applied to yearly data confirm significant increasing trends for both compound hazard categories (1 compound hazard: *P* = 0.0024, *τ* ≈ 0.33; 2 + compound hazards: *P* = 0.0025, *τ* ≈ 0.33), whereas single-flood events showed no significant trend (*P* = 0.61, *τ* ≈ 0.06). The data further indicate an overall increase in the proportional share of compound events, alongside a relative decline in single-flood events. Together, these findings suggest a long-term increase in the frequency of floods associated with compound hazards across Europe, consistent with broader hydro-climatic changes^47–51^.

To examine the relationship between compound hazard types and flood impact severity, we group events into five impact classes based on reported economic losses in Fig. 2b. Consistent with previous studies^7,12,52^, the distribution of losses is strongly heavy-tailed. Figure 2b highlights a pronounced shift in event composition: compound events become increasingly dominant in higher-impact classes, while single floods nearly vanish and are entirely absent from the top 1% of losses. The escalation of losses is markedly stronger for compound events: from the lower 50% to the top 10% impact class, “single-flood” events show roughly a 100-fold increase, “1 compound hazard” events rise nearly 1000-fold up to the top 1%, and “2 + compound hazards” events increase about 400-fold (Fig. 2b). Total recorded losses amount to ~19.3 billion euros for “single-flood” events, 73.7 billion for “1 compound hazard” events, and 112.7 billion for “2 + compound hazards” events. The most damaging compound events produce, on average, around 11 billion euros more losses than the largest-loss single floods, highlighting that compound events disproportionately drive the largest economic impacts. Statistical significance was evaluated using a permutation test (Fig. 2d), randomly reshuffling flood losses to compare the observed mean differences. Losses for “1 compound hazard” events are significantly higher than for “single-flood” events (*P* = 0.0126), and “2 + compound hazards” events show an even stronger difference (*P* = 0.0002). As larger floods are more likely to be categorized as compound by construction, we use footprint-stratified analyses to disentangle spatial extent from compound amplification. We find that compound effects are most pronounced for localized floods—where overlapping hazards characterize a larger share of the affected area—whereas in very large floods, the signal may be spatially diluted and the total impact increasingly dominated by footprint (Supplementary Fig. 2 in the Supplementary Information file).

### Flood losses are shaped by compound hazard complexity

To assess the influence of compound hazard complexity on flood losses, we employed an ensemble Gradient Boosted Machine (GBM) framework to model the mean log-transformed flood damages per NUTS3 region. Some NUTS3 regions share the same flood event, which could introduce minor dependence across observations; averaging losses within regions reduces this effect. Predictor variables were aggregated at the administrative level using appropriate statistical summaries, as detailed in the “Methods”. These included temporal lags between flood-related hazards and event onset, the total number and intensity of associated hazards, flood magnitude (expressed as return periods)^53,54^, and population and built-up area metrics from the Global Human Settlement Layer (GHSL)^55^. Socioeconomic indicators of vulnerability, such as the Social Vulnerability Index (SoVI)^56^ and the Risk Data Hub Vulnerability Index (RDH VI)^57^, were incorporated alongside combined exposure-vulnerability metrics including gridded GDP, Human Development Index (HDI), and the Gini coefficient^58^. Among all predictors, log-transformed flood loss showed the strongest correlation with compound hazard complexity (24.5%), supporting its relevance for modeling. Details on selected and harmonized predictors, as well as a full correlation matrix, are available in Methods (see also Supplementary Fig. 11 in the Supplementary Information file). The models achieved an average *R* of 0.47, indicating moderate predictive performance, with bootstrapping applied across NUTS-3 regions to ensure robustness (Supplementary Fig. 12).

**Fig. 3:**
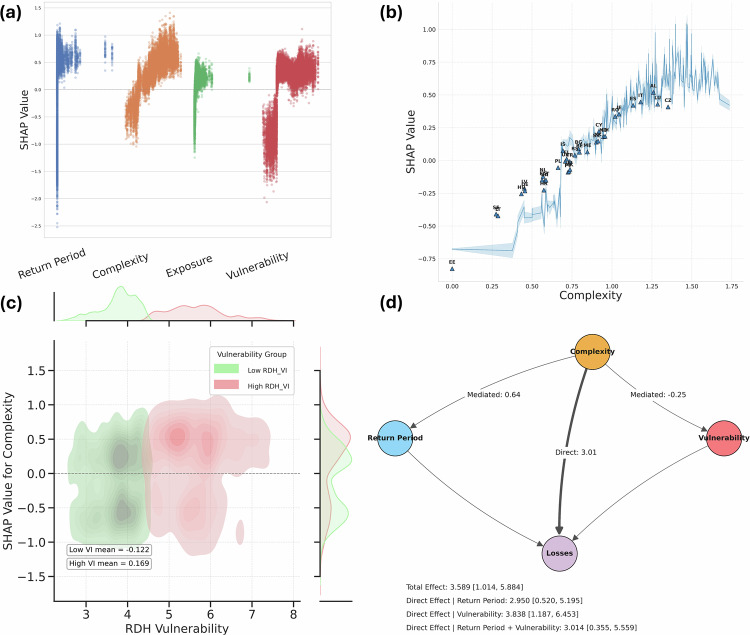
SHAP analysis of drivers of flood losses, the role of compound hazard complexity, and the estimated average treatment effects obtained from Causal Forests. **a** Relationship between SHAP values and normalized feature values for key predictors: return period, complexity, exposure defined by built-up area, and RDH vulnerability index. **b** SHAP-feature relationship for complexity with overlaid country-level means, illustrating national contexts within the compoundness-impact space. **c** Distribution of SHAP values for complexity stratified by low (< 4.5) and high (> 4.5) vulnerability, indicating stronger effects under high vulnerability conditions. **d** Directed acyclic graph illustrating the decomposition of the effect of compound hazard complexity on flood losses into direct and mediated pathways through return period and vulnerability. Edge labels indicate estimated effect sizes. Node colors represent the components of the causal structure: complexity (orange), flood magnitude/return period (light blue), RDH vulnerability (red), and log-losses (purple).

To understand the influence of compound hazard interactions on predictions, we applied SHAP (SHapley Additive exPlanations), which allowed us to attribute model predictions to specific features and their interactions^59^. SHAP values quantify how much each feature increases or decreases the predicted flood losses compared to the mean prediction. Positive SHAP values indicate that a feature increases the prediction above the mean, suggesting a higher risk of loss, while negative SHAP values indicate a reduction in predicted losses. Importantly, SHAP values quantify associations within the predictive model and should not be interpreted as causal relationships. They indicate how the model’s internal structure attributes variation in predicted losses to each feature. The models robustly capture expected relationships between hazard magnitude, exposure, vulnerability, and damages (see Fig. 3a). Most importantly, the SHAP analysis shows that compound hazard complexity is a key driver of predicted flood losses: higher complexity values are associated with larger positive SHAP contributions (i.e., increases in the model’s predicted log losses). Considering both SHAP and permutation importance (see Supplementary Fig. 13 in the Supplementary Information file), complexity consistently ranks among the top five predictors and accounts for ~10% of the total absolute SHAP magnitude. For additional context, mean SHAP values aggregated at the country level are overlaid on the scatter plot to provide a broad indication of national patterns, despite the analysis being conducted at a subnational resolution (Fig. 3b). The SHAP analysis indicates that complexity contributes positively to predicted losses when regions experience at least two or more distinct event types (see Fig. 3b). See also the full SHAP diagram in Supplementary Information Figure 13a (see Supplementary Information file). Vulnerability indicators (Social Vulnerability Index and Risk Data Hub Vulnerability Index) jointly contribute to 20% of average absolute SHAP values (see Supplementary Fig. 13c), emphasizing the role of social fragility. Exposure and downscaled socioeconomic metrics each contribute roughly 10%. Individual compound event counts have a limited role, as their effects are largely captured by the complexity metric (see again the correlation matrix in Supplementary Fig. 11). Stratifying by vulnerability (RDH-VI) shows that the SHAP effect of complexity exhibits opposite signs in low-vulnerability regions (RDH-VI <4.5) versus high-vulnerability regions (RDH-VI >4.5), indicating that higher complexity contributes differently to predicted losses depending on local vulnerability levels (Fig. 3c; full analysis in Supplementary Fig. 14 in the Supplementary Information file). Additional details on the model training, validation procedures, and full SHAP rankings are provided in the “Methods” and Supplementary Information Sections.

To empirically assess whether hazard complexity directly affects flood losses, we estimated total and direct effects using a Double Machine Learning (DML) Causal Forest^45,60^, controlling for all other covariates (Fig. 3d). The total effect of complexity (ATE = 3.589, 95% CI [1.014, 5.884]) captures all pathways, including potential associations with flood magnitude (i.e., Return Period) and vulnerability (i.e., RDH-VI). Controlling for magnitude reduces the effect slightly (ATE = 2.950, 95% CI [0.520, 5.195]), indicating that differences in flood return period explain only a limited portion of the observed association. Controlling for vulnerability yields a similarly stable estimate (ATE = 3.838, 95% CI [1.187, 6.453]), further supporting that the complexity-loss relationship is robust to adjustment for RDH-VI under our modeling framework. When controlling for both pathways, the effect remains positive and substantial (ATE =  3.014, 95% CI [0.355, 5.559]). To further clarify the relationship between complexity and flood return period, we explicitly tested bidirectional causal effects using two additional DML models (i.e., complexity  → return period and return period  → complexity). Both Average Treatment Effects were approximately three to four times smaller than the complexity-loss effect. Overall, these results indicate that the amplification of losses associated with compound hazard complexity is largely independent of return period and unlikely to be substantially driven by confounding through hazard magnitude. While the analysis does not identify the precise mechanism behind this effect, a plausible interpretation is that greater heterogeneity associated with a higher mix of hazard configurations may contribute to increased impacts. A summary of the tested causal pathways and main results of the DML analysis is provided in Fig. 3d.

## Discussion

This study provides data-driven evidence that the diversity of compound events amplifies flood losses across European regions. We observe that compound hazard events have increased substantially over the past four decades, both in absolute and proportional terms. Examining loss distributions, permutation tests indicate that compound events are associated with significantly higher economic impacts than single floods, particularly for the most damaging events. By applying machine-learning approaches—including GBM with SHAP explanations and Causal Forests to estimate average treatment effects at the NUTS3 regional level—we show that compound hazard complexity, along with higher flood magnitude and regional vulnerability, consistently contributes to elevated flood losses. Taken together, the temporal trends, loss distributions, and ML analyses provide convergent evidence that compound hazard complexity plays an important role in shaping flood impacts. This underscores the need for risk management strategies that account for multi-hazard interrelations, besides the spatial distribution of exposure and vulnerability.

The analysis is constrained by the limited size and spatial resolution of the available historical data. While the HANZE dataset provides event-level flood losses aggregated at the NUTS-3 level, more advanced disaggregation or downscaling techniques could be employed to capture local variability. Causal Forests provide estimates of average treatment effects, but these should be interpreted cautiously, as they rely on model assumptions and observed variables and do not alone establish definitive causal relationships. A natural next step would be to make use of climate model projections to extract from hydrometeorological hazard events future scenarios, enabling forward-looking assessments of how compounding hazards may influence flood risks in a warming climate. Our modeling framework focuses on average flood loss patterns at the NUTS-3 level to identify broad spatial relationships, but this regional scale may overlook important sub-regional variations in exposure and impact, which is also reflected in the limited predictive performance of the trained models. Similarly, while vulnerability indices offer subnational detail, they remain largely static and may not capture dynamic changes over shorter time scales-such as those linked to recovery processes or evolving adaptive capacity. As more detailed and time-resolved hazard and impact datasets become available, it would be valuable to extend this approach to better represent temporal variability and more localized risk patterns.

Future research should examine how different economic sectors respond to compound hazard impacts relative to single hazards, for example, agricultural losses driven by heatwave-flood or drought-flood transitions, versus infrastructure damages linked to wind-flood events. Disaggregating impacts by sector will be critical to identify which compound event archetypes pose the greatest risks and to design sector-specific resilience strategies. Developing dynamic vulnerability models that reflect socioeconomic and environmental changes over time is also essential to support anticipatory policy planning. While our study links compound events, historical flood losses, and societal vulnerability at a broad scale, it does not resolve the underlying mechanisms in each context. Translating compound hazard typologies into targeted policy actions, such as improving water retention in drought-prone regions or enhancing wind resistance in coastal zones, can enhance the effectiveness of disaster risk reduction strategies but requires additional efforts in data collection. Embedding compound hazard perspectives into national risk assessments, adaptation planning, and disaster resilience goals will be increasingly necessary as climate extremes intensify. In this context, strengthening multi-hazard risk management approaches that account for complexity, interdependencies, and sectoral sensitivities is not only scientifically sound but also a policy imperative.

## Methods

### Hydrometeorological hazards and flood impact datasets

Flood impact records were obtained from the HANZE database- the most comprehensive record of flood-related disaster losses available for the continent^29,30^. HANZE harmonizes 2521 riverine, flash, and coastal flood events across 42 European countries for 1870–2020—with detailed spatiotemporal and impact information. These events are compiled from a large set of historical and contemporary documentary sources, national flood catalogs, civil protection reports, and scientific literature, with the full list of data sources publicly available (https://naturalhazards.eu/list-of-references). The underlying data are known to be uneven in space and time: coverage is relatively comprehensive after 1990, while earlier decades—particularly in parts of Eastern Europe—show lower reporting density^53^. A chain of hydrological and impact models, based on the HERA hydrological reanalysis^61^, has been developed to recreate riverine, coastal, and compound floods with potential socioeconomic impacts across Europe for the period 1950–2020. This allowed for the validation of historical floods in HANZE, showing that the dataset captures ~92% of total flood economic losses over this period^53^, indicating high completeness, especially during the study period from 1981 to 2020. These records include data on inundated areas, fatalities, affected persons, and economic losses, as well as the administrative units affected by each event, at the NUTS-3 level (Nomenclature of Territorial Units for Statistics, level 3), which corresponds to small regions used for statistical reporting in the European Union. The substantial variation in area and population across NUTS-3 regions can influence the meeting of regional-scale impact thresholds. This variability is a known source of uncertainty in HANZE, as discussed in the literature^53^. For this study, we focus on flash and riverine flood events occurring between January 1, 1981 and December 31, 2020, retaining for each event its geographical location, start date, and reported economic loss. We exclude compound river-coastal events because the relative contribution of fluvial and coastal forcing varies substantially between events, complicating attribution and inhibiting comparable predictor construction in our compound-hazard modeling framework. To harmonize the analysis, all reported economic losses from HANZE were uniformly distributed across the affected NUTS-3 region. While this approach does not account for local variations in exposure, it facilitates consistent aggregation and interpretation at the subnational level. Additionally, equal distribution was necessary to avoid circular reasoning in the subsequent modeling of losses, where variables such as exposure and return period are used as predictors. Events with “no recorded impacts” in HANZE do not represent true zero-loss events; rather, they indicate missing economic-loss data for events that met minimum inclusion criteria through other impact indicators (e.g., fatalities, persons affected, or inundated area). Model-based analysis shows that such events tend to have economic losses approximately three times smaller, on average, than events with reported damage, consistent with their partial but non-zero socioeconomic impact^62^.

For each associated hazard, we derive impact-relevant metrics. Percentile-based threshold approaches were used to identify extreme wind, high, and low flows. For heatwaves and coldwaves, we used the Heat and Cold Wave Index (HCWI)^63^, which captures the persistence and intensity of temperature anomalies sustained over consecutive days. Associated hazards were obtained from (i) climate reanalysis (ERA5 and ERA5 Land), (ii) hydrological reanalysis (HERA)^61^, and (iii) interpolated observational datasets (MARS AGRI4CAST/EDO Heat and Cold Wave Index). We justify this diversity of inputs by our desire to build on existing hazard-related European data products. Extreme wind data were retrieved from the ERA5 10 m instantaneous wind gust variable. Initially, we aggregated the data spatially by taking the maximum wind speed over each NUTS-3 region’s geographical area. Subsequently, we identified temporal anomalies by determining occurrences that exceeded the 99.5th percentile of the all-season distribution across the entire domain. This method ensures that both spatial and temporal extremes are effectively captured in our analysis. For drought and flood hazards, we relied on the HERA high-resolution hydrological reanalysis^61^ generated with the OS LISFLOOD hydrological model driven by bias-corrected, downscaled ERA5 Land forcings. HERA includes river discharge data at 282,000 river pixels with an upstream area greater than 100 km^2^ in Europe for the period 1951–2020. Discharge data was aggregated from the native pixel resolution (1 arcminute) to the NUTS3 level by averaging the specific discharge (m^3^/s/km^2^) of every river pixel in a given NUTS3 region. For high flows (floods), we first extract the daily maximum specific discharge at the regional level (Qmax). Hazard events were detected using a peak-over-threshold approach using the local 98th percentile (computed over the entire study period) as a threshold for extreme event detection. For low-flow (drought) events, discharge data were temporarily aggregated to a 7-day moving average (Q7), a frequently used metric for drought frequency analysis^64^. We used a monthly (30-day rolling window around each calendar day) variable threshold level approach^65^. Extreme events were detected when Q7 fell below the 5th percentile of all Q7 values recorded in a 30-day rolling window around a specific day of the year. Following a pooling strategy, consecutive low-flow episodes separated by fewer than 30 days were merged into a single drought event. A minimum duration threshold of 10 days was then applied to ensure that only sustained droughts were retained. Heat and cold waves were identified via the Heat Cold Wave Index (HCWI): daily maximum (Tmax) or minimum (Tmin) temperatures persisting above the 90th or below the 10th percentile, respectively, for ≥3 consecutive days, with percentiles computed on a 30-year (1981–2010) climatology from the JRC MARS AGRI4CAST database^63^. Maps of NUTS3-level occurrences for all five hazards are shown in Supplementary Figs. 6, 7, and 8, while Supplementary Figs. 3 and 4 present the analysis of temporal trends (see Supplementary Information file).

The flood return periods were calculated based on a hydrological reanalysis covering the period 1950–2020, described and validated by Tilloy et al.^61^. It provides daily river discharge time series for the entire domain at 1.8 km spatial resolution. Transformed-stationary extreme value analysis^66^ was used to detrend the non-stationary grid-cell discharge time series and to calculate the stationary return periods by fitting a generalized Pareto distribution to the peaks over a threshold. We obtained the return period of an event as the geometric average of the return periods of river grid cells contributing to the event^53^. Because not all observed flood events have a corresponding modeled discharge anomaly within the hydrological reanalysis domain, some return-period values were missing^53^; these were imputed by first replacing missing values with the mean return period of events within the same NUTS3 region and, if still unavailable, with the mean within the same event. This procedure reduces within-group variability and may therefore lead to a conservative (i.e., potentially underestimated) estimate of the predictive importance of return period.

### Creation of compound events related to flood impacts

A compound event was recorded when an associated hazard (drought, flood, heatwave, coldwave, or windstorm) occurred within a defined temporal window relative to the start date of a flood, and both events fell within the same NUTS3 region. Defining an appropriate window for compound event analysis is complex^20^, so we considered three temporal windows: short (14 days), medium (28 days), and long (56 days). We computed the temporal distance between the start and end dates of the associated hazard events and recorded flood events. Compound events were identified if an associated hazard event ends a given number of days before the beginning of the flood record (depending on the window). With the exception of wet sequence, which by nature has to happen in succession, we also identified compound events if a hazard event starts during the recorded flood event. For the subsequent analysis, we applied a literature-informed window, as detailed in Table 1, to effectively capture compound events. In Supplementary Information Fig. 1 (see Supplementary Information file), we show how the distribution of compound events, including single floods, changes across different temporal overlap windows (short = 14 days, medium = 28 days, long = 56 days). As the window length increases, the share of compound events rises. We examined five compound hazard types: (1) wet sequences, (2) drought flood events, (3) heat-flood sequences, (4) cold-flood events, and (5) wind-flood events. For each recorded flood event, we counted the number of associated hazard types occurring within the defined temporal window and within the same NUTS3 region. Floods with no associated hazard types were classified as “ single-flood” events. Floods associated with exactly one hazard type were classified as “ 1 compound hazard” events, while floods associated with two or more hazard types were classified as “ 2 + compound hazards” events. This approach avoids duplicating flood records and ensures that each event is assigned to a single, mutually exclusive category. The raw results of the temporal matching and hazard-type classification are shown in the Supplementary Information Fig. 1 and Supplementary Tables 1, 2, 3 (see Supplementary Information File). We provide here a short description of each flood-related compound hazard type.

Wet sequences are periods of extremely high-flow events that precede flood impacts, often caused by persistent atmospheric conditions like extratropical cyclones or atmospheric rivers, particularly in Western Europe^31^. This hazard interrelation falls within the precondition and temporally compounding types^2^. These sequences can last from weeks to months, depending on storm duration and catchment properties, and can be connected to riverine or flash floods. Notable examples include the winter 2014 storms in the British Isles. Wet sequences can lead to increased flood severity and damage, as regions hit by a flood can still be recovering from previous floods, increasing vulnerability and reducing the community’s capacity to cope^67^. We identify wet sequences using NUTS3 aggregated high-flow anomalies rather than precipitation, as streamflow integrates antecedent storage and soil moisture, which are critical for flood generation^33^. According to previous research^31,32^, we identify wet-sequences if previous high flow anomalies occurred between 4 and 56 days prior to the flood impact event, with the 4-day lower bound ensuring that only antecedent conditions, and not the flood itself, are captured.

Drought-flood events occur when floods follow or coincide with droughts, driven by transitions from dry to wet conditions^36^. Drought-induced dry soil can lead to increased runoff during precipitation events^36^, causing flash floods. Drought-flood events are multivariate or temporally compounding events^2^ as these events often span long durations, with floods occurring during or up to 2 months after a drought^68^. Existing drought impacts can worsen flood damage, affecting infrastructure and agricultural practices^64^. The 0–56 day window is designed to capture rapid transitions from dry to wet conditions, including floods occurring during the late stages of a drought or shortly after it ends, when antecedent soil dryness maximizes runoff^35^. Drought-flood events are detected if low flow anomalies occurred up to 56 days prior to the flood impact event.

Hot–wet sequences involve floods following heatwaves and are often linked to thunderstorms^37^. Triggered by hot, humid conditions, these events are mostly associated with flash floods. This hazard interrelation is therefore of the precondition type^2^. Typically developing within 3–14 days, they can stress health infrastructures and disproportionately affect vulnerable populations^69^. Socioeconomic factors further exacerbate these impacts, with poorer populations being more vulnerable to the compounded effects of heatwaves and floods^37^. Hot–wet events are detected if a heatwave occurred up to 14 days prior to the flood impact event.

Compound cold and flood events involve floods during or after cold waves, often linked to snowmelt, ice jams, or fluvial floods^70,71^. A rapid shift from cold to mild conditions is required to trigger these floods. This hazard interrelation can be classified as the precondition type^2^. Such events can damage agriculture if occurring during growing seasons, and can stress health systems^72^. Although not as well defined as other multi-hazard events, these events are crucial due to their association with snow processes, particularly snowmelt, which is a well-known flood mechanism in Europe^31,73^. Compound cold and flood events are detected if a coldwave occurred up to 14 days prior to the flood impact event.

Compound windstorm and flood events, prevalent in Western Europe, are driven by extratropical cyclones, causing simultaneous extreme wind and precipitation^74^. The interrelation between flood and wind can be multivariate or temporally compound and lead to flash, fluvial, or groundwater floods, with impacts lasting from hours to days. Such events are of interest to sectors like insurance and energy due to their potential for compounded damages^42^. These events are particularly concerning during winter, when extratropical cyclones frequently strike Europe in sequences, leading to increased risks of wet sequences^75^. Transport infrastructure such as road and rail networks can be severely impacted, leading to cascading effects throughout the region^18,67^. As extratropical cyclones can occur in sequences, we consider that compound windstorm-flood events occur if a flood impact event co-occurs or is preceded by extreme wind in the previous 56 days.

Total observations of these compound hazards at NUTS3 level are then summarized in a single index. In particular, we introduce the compound hazard complexity (or simply complexity) as the Shannon entropy: $\,{\rm{CC}}\,=-{\sum }_{i=1}^{M}{p}_{i}ln({p}_{i})$, where *p*_*i*_ is the fraction of occurrences of type *i* and *M* is the total number of distinct types observed in a given NUTS3 region. This metric captures the mix of compound hazards associated with HANZE flood impacts.

### Temporal trend and permutation tests

We assessed long-term trends in yearly flood and compound event counts using the Mann–Kendall non-parametric test for monotonic trends. The Mann–Kendall test evaluates whether there is a consistent increase or decrease in a time series without assuming any specific distribution of the data. For a series of *n* observations, the test compares all pairs of observations (*x*_*j*_, *x*_*k*_) with *j* < *k*, counting the number of times a later observation is greater than an earlier one and the number of times it is smaller. The test statistic *S* is the difference between these counts. Kendall’s *τ* is then computed as a normalized measure of correlation between the ranks of the data, ranging from −1 (perfect decreasing trend) to +1 (perfect increasing trend). The significance of the trend is determined by the *P* value associated with *S*, calculated under the null hypothesis of no trend. To compare flood loss distributions across event types in a model-independent manner, we employed permutation tests. For each test, the log-transformed losses were randomly reassigned among the categories 10,000 times to create a null distribution of mean differences between categories, representing the differences expected under the null hypothesis of no association. The *P* value was calculated as the proportion of permutations for which the mean difference exceeded the observed mean difference in absolute value, providing a non-parametric measure of statistical significance. This approach does not rely on any assumptions about the underlying data distribution or variance, making it particularly suitable for heavy-tailed or skewed loss distributions. Permutation tests were applied to assess whether floods with one or more compound hazards exhibit systematically higher losses than single-flood events.

### Vulnerability and exposure indicators

To characterize vulnerability, we utilized two complementary subnational indices. The first, the Risk Data Hub Vulnerability Index (RDH VI) by the JRC, is a composite indicator assessing vulnerability at national and subnational scale (NUTS-2 and NUTS-3 levels)^57^. It integrates five dimensions-social, economic, political, environmental, and physical-using 47 indicators at multiple administrative levels. Indicators are normalized and aggregated through a hierarchical, non-compensatory approach (Adjusted Mazziotta–Pareto Index) to produce a robust, spatially and temporally consistent index^57^. For our study, we averaged RDH VI over time at the NUTS-3 level to match the static nature of regional economic loss values. Its multidimensional design captures key vulnerability drivers, making it suitable for modeling flood losses by reflecting the broader context in which hazards translate into economic impacts. The second, the global Social Vulnerability Index (Globe-SoVI), is derived from an analysis of 913 historical flood events and identifies five key social vulnerability drivers: mean years of schooling, elderly population share, gender income gap, rural settlement prevalence, and walking time to healthcare^56^. These variables were weighted using regression models to determine their impact contributions. The SoVI is static and provides a spatially detailed (1 km resolution) and flood-related portrayal of vulnerability, which we averaged over each NUTS-3 region for our analysis. Although developed at the global scale, its consistent methodology and openly available input datasets make it directly applicable to the European context. Importantly, while GlobE-SoVI is designed to model flood-related fatalities, the same underlying social characteristics-such as education, income inequality, age structure, and access to health services-are known to influence economic loss potential through channels including preparedness, preventive maintenance, institutional response capacity, and recovery speed. The downscaled datasets for GDP, HDI, and Gini are created using machine-learning techniques to predict missing data and increase detail^58^. This process improves the datasets’ resolution to a finer scale, reaching administrative level 2, for the years 1990 to 2022. These downscaled variables are instrumental in characterizing vulnerability, while also accounting to some extent for exposure, as factors such as population density and urbanization levels are integrated into their construction. This aligns with studies showing that higher wealth and development generally reduce vulnerability to natural hazards^76–79^. To ensure compatibility with HANZE flood records, we adjusted the reference system by aligning the maximum intersection of Global Administrative Areas (GADM) level 2 with EU NUTS-3 regions. We also utilized exposure variables derived from the Global Human Settlement Layer (GHSL) developed based on Sentinel-2, Landsat, and global DEM data^55^. This dataset offers multitemporal estimates of both population and built-up volumes, including residential and non-residential uses, from 1975 to 2030 at 5-year intervals. To ensure consistency with the rest of the data, we averaged the 100-meter resolution variables at the NUTS-3 level. Subsequently, we averaged the NUTS-3 values for both exposure and vulnerability variables across the entire temporal period considered in the study.

### Explainable machine-learning modeling with SHAP values and causal forests

Each instance in our dataset represents a NUTS-3 region, with all quantities aggregated at this subnational level, as detailed in previous sections. To ensure comparability across variables, we normalized the data by subtracting the minimum value and dividing by the range, rescaling each feature to a 0 to 1 range. Missing values were imputed with zeroes to maintain dataset integrity. Additionally, average population data underwent log transformation to address skewness, and the target variable representing flood losses was similarly log-transformed to promote a more normal distribution of residuals during modeling. Missing values for the return period were filled by first replacing them with the mean return period within each NUTS-3 group. For predicting flood losses, we employed Gradient Boosting Regression alongside a comprehensive evaluation strategy involving 300 random train-test splits. In each iteration, the dataset was divided into 90% training and 10% testing sets, allowing for a robust assessment of the model’s predictive capabilities despite the limited sample size (see Supplementary Information Fig. 12 in the Supplementary Information file). We reserved 10% of the training set for cross-validation, optimizing hyperparameters. To identify the best hyperparameters, we conducted a randomized search over a predefined parameter space. The parameters explored included the number of estimators ([50, 120]), maximum depth of trees ([2, 3, 4]), minimum samples required to split a node ([12, 24]), minimum samples required at leaf nodes ([10, 16]), learning rate ([0.01, 0.05, 0.1]), maximum features considered for splitting ([6, *sqrt*, *log*2]), and subsample fraction ([0.7, 0.9]). This search evaluated multiple combinations to balance thoroughness and computational efficiency. The process included cross-validation to ensure robust performance evaluation, leveraging parallel computing to expedite the search. The final model for each iteration, trained with these optimal parameters, was evaluated using mean squared error (MSE) and R-squared metrics for both training and testing datasets, ensuring accuracy and reliability. As machine-learning models become larger and more complex, understanding their prediction mechanisms has become increasingly challenging. In response, the field of Explainable AI offers a range of methods to enhance interpretability. These include post-hoc techniques like feature ranking and approaches designed for inherent interpretability. One widely used method for interpreting model predictions is based on Shapley Value sampling theory. Originally from cooperative game theory, Shapley values allocate a model’s output among its input features, providing a local explanation for individual predictions^59^. The Shapley value for each feature is calculated as the average of its marginal contributions across all possible feature permutations, using approximation schemas to reduce computational cost. SHAP values were computed for each model iteration. These values were averaged to provide insights into feature importance at the NUTS-3 level across the dataset. This approach not only ensured predictive accuracy but also improved transparency in the model’s outcomes. Additional explainability analyses are presented in Supplementary information Fig. 13 (see Supplementary Information).

We estimate the causal effect of flood complexity on (log-transformed) mean losses using a double machine learning (DML) framework with causal forests^45^, implemented via the EconML package^60^. Causal forests extend the random forest methodology to estimate heterogeneous treatment effects by predicting potential outcomes under treatment and control for each observation. The average treatment effect (ATE) quantifies the expected change in the outcome induced by a unit-level change in the treatment, holding confounding covariates constant. In our analysis, the treatment of interest is flood complexity, scaled between 0 and 1, and confounders include pre-event hazards, socioeconomic indicators, exposure, and vulnerability metrics. We employ a repeated-sample DML strategy, where gradient boosting models, configured with parameters such as a learning rate of 0.05 and a maximum depth of 3, estimate the nuisance functions for both outcome and treatment. Causal forests, with 400 estimators and a minimum sample leaf of 5, then estimate the ATE across 300 train-test splits. This approach allows robust estimation while mitigating bias from high-dimensional confounders. To distinguish total and direct effects of complexity, we vary the set of mediators included as confounders: (i) for the total effect, mediators such as return period and vulnerability indices are excluded from confounders; (ii) for direct effects, one or both mediators are retained as confounders to isolate the portion of the complexity effect not transmitted through these channels. Confidence intervals for the ATE are obtained from the empirical distribution across splits. Our analysis assumes no unmeasured confounders (or causal sufficiency hypothesis)^80^, meaning all relevant variables are included, which is critical for valid causal inference. Any omitted confounder could bias results. Causal interpretation requires careful control of potential confounding factors. Even when using formal causal inference methods, recent studies demonstrate substantial challenges in recovering true causal links, including limitations in both precision and recall. Consequently, causally inferred links should be interpreted with caution-they may increase confidence in the robustness of detected associations, but do not, on their own, establish definitive causal relationships. While causal inference methods provide a more rigorous framework for evaluating statistical associations in the presence of potential confounders, they also have well-documented limitations in the literature^80–83^.

## Supplementary information

Supplementary Information

Peer Review file

## Data Availability

All data needed to support the conclusions in the paper are freely available in Zenodo at 10.5281/zenodo.19568456.

## Code availability

The code and some demos containing our experiments can be found in the GitHub repository at https://github.com/Alowis/CFRisks_codes/tree/mib and a permanent version in Zenodo at 10.5281/zenodo.19568456.
