# Supplementary material for: Compounding hazards increase flood economic losses across Europe
Source: Nat Commun. 2026 May 19;17:6614. doi: 10.1038/s41467-026-73248-0 (PMC13381573; doi:10.1038/s41467-026-73248-0)
Supplement: Supplementary file 1 — Supplementary Information [file 41467_2026_73248_MOESM1_ESM.pdf]

Supplementary information

Events distribution

HANZE floods affecting one or more NUTS-3 regions are matched to the NUTS-3 hazard time series using four temporal window options: informed, short (14 days), medium (28 days), and long (56 days).Figure 1b shows how the distribution of events across the three categories varies for four temporal windows (short, medium, long, and informed), illustrating the effect of window choice on the classification. For each flood, all associated hazard–flood pairs across affected NUTS-3 regions are recorded; for example, a flood affecting NUTS A and B may coincide with a heatwave in A and a drought in B. Supplementary Table 1 reports the counts of these overlapping hazard–flood pairs, providing full transparency of the sample. These overlapping pairs are then mapped into three mutually exclusive event-level categories: "single-flood", "1 compound hazard", and "2+ compound hazards" (Table 2). Each flood is assigned to exactly one category based on the total number of associated hazards, ensuring that no event is double-counted. Supplementary Table 3 summarises the composition of these categories in terms of the contributing hazard types. Supplementary Figure 1a further illustrates the distribution of all possible hazard combinations, highlighting that single-flood events dominate while compound events are dispersed across diverse combinations.

To investigate the influence of flood size on event losses, we stratified events by the number of affected NUTS-3 regions (flood footprint). Larger floods are inherently more likely to overlap with additional hazards, resulting in a physical coupling between flood extent and compound classification (Fig. 2a). Consequently, single-flood events are scarce at larger scales ($N = 26$ for ≥ 3 regions). For spatially limited floods (1–2 regions), compound events exhibit significantly higher log-losses than single-flood events ($p = 0.048$; Fig. 2b), as assessed with a one-sided random permutation test (10^4 reshufflings) evaluating whether the median loss of compound events exceeds that of single floods. While statistical power is limited for larger floods ($\geq$ 3 regions; $p = 0.63$), median trends remain consistent. When losses are normalized by the number of affected regions, compound events—particularly those with 2+ additional hazards—show higher per-region impacts ($p = 0.033$; Fig. 2c). This suggests a dilution effect at larger scales: localized floods are more likely to be compound across their entire footprint, while larger floods may be dominated by regions experiencing only single-hazard conditions. In Fig. 2 the box spans the interquartile range (IQR), the horizontal line denotes the median, whiskers extend to $1.5 \times$ IQR.

**Compound Hazard–Flood Pair**	**Count**
Wet-sequence	590
Single-flood	390
Windstorm–flood	379
Drought–flood	290
Heat–flood	122
Cold–flood	66

Supplementary Table 1: Counts of Overlapping Compound Hazard–Flood Pairs

**Category**	**Count**
Single-flood	390
1 compound hazard	553
2+ compound hazards	406

Supplementary Table 2: Distribution of Mutually Exclusive Event-Level Categories

**Category**	**1 compound hazard**	**2+ compound hazards**	**Single-flood**
Drought-flood	42.1	57.9	0.0
Wet-sequence	45.4	54.6	0.0
Heat-flood	42.6	57.4	0.0
Cold-flood	22.7	77.3	0.0
Windstorm-flood	25.3	74.7	0.0
Single-flood	0.0	0.0	100.0

Supplementary Table 3: Distribution of Compound Hazard Pairs (%)

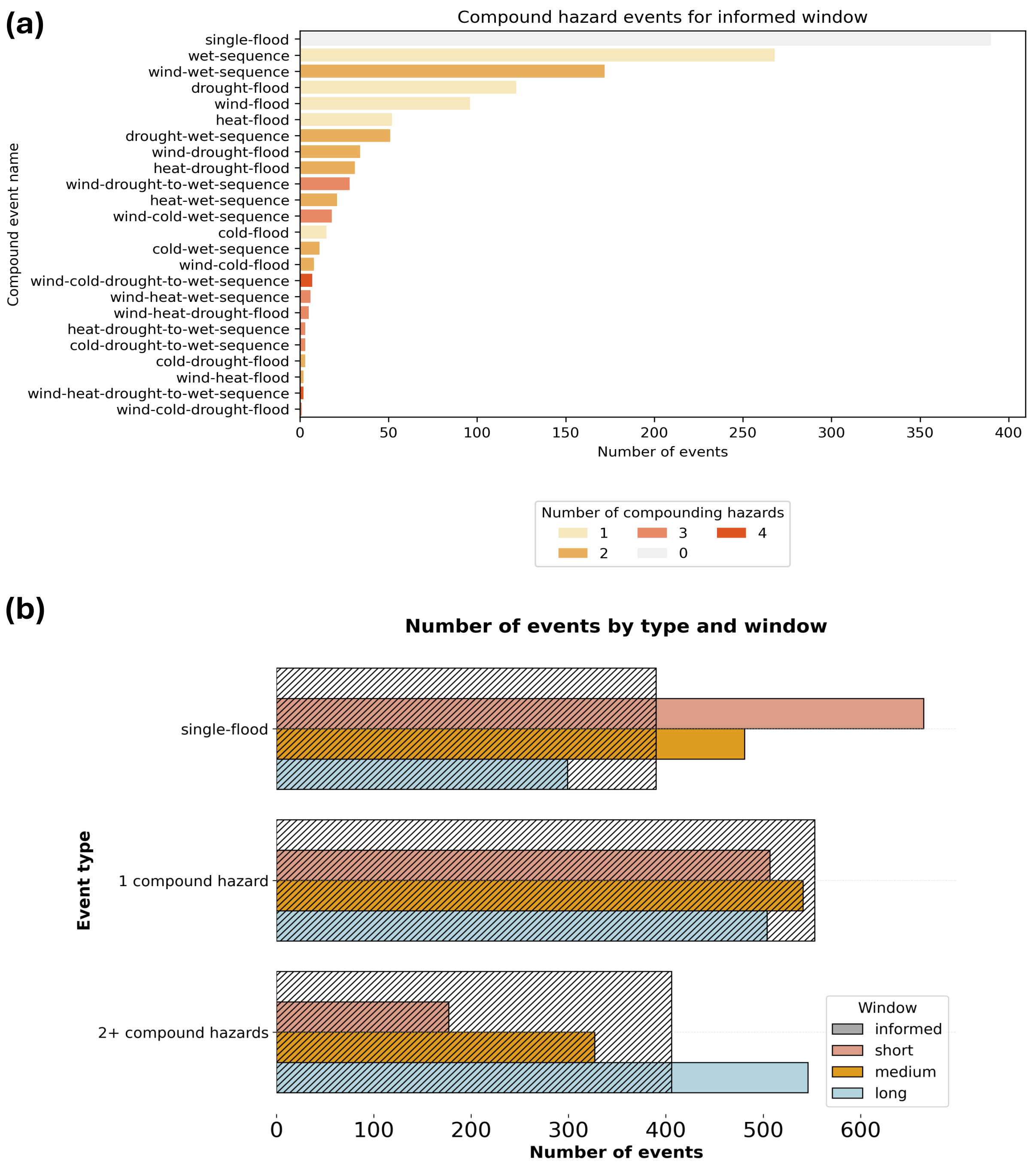

Supplementary Figure 1: Distribution of flood events and compound hazard classifications under different temporal windows. (a) Distribution of event types, including multiple compound hazards, illustrating observed event chains such as wind, heat, wet, and flood according to our methodology for compiling compound events. (b) Number of records per event type, i.e. “single-flood”, “1 compound hazard”, and “2+ compound hazards”, analyzed using informed, short (14 days), medium (28 days), and long (56 days) windows.

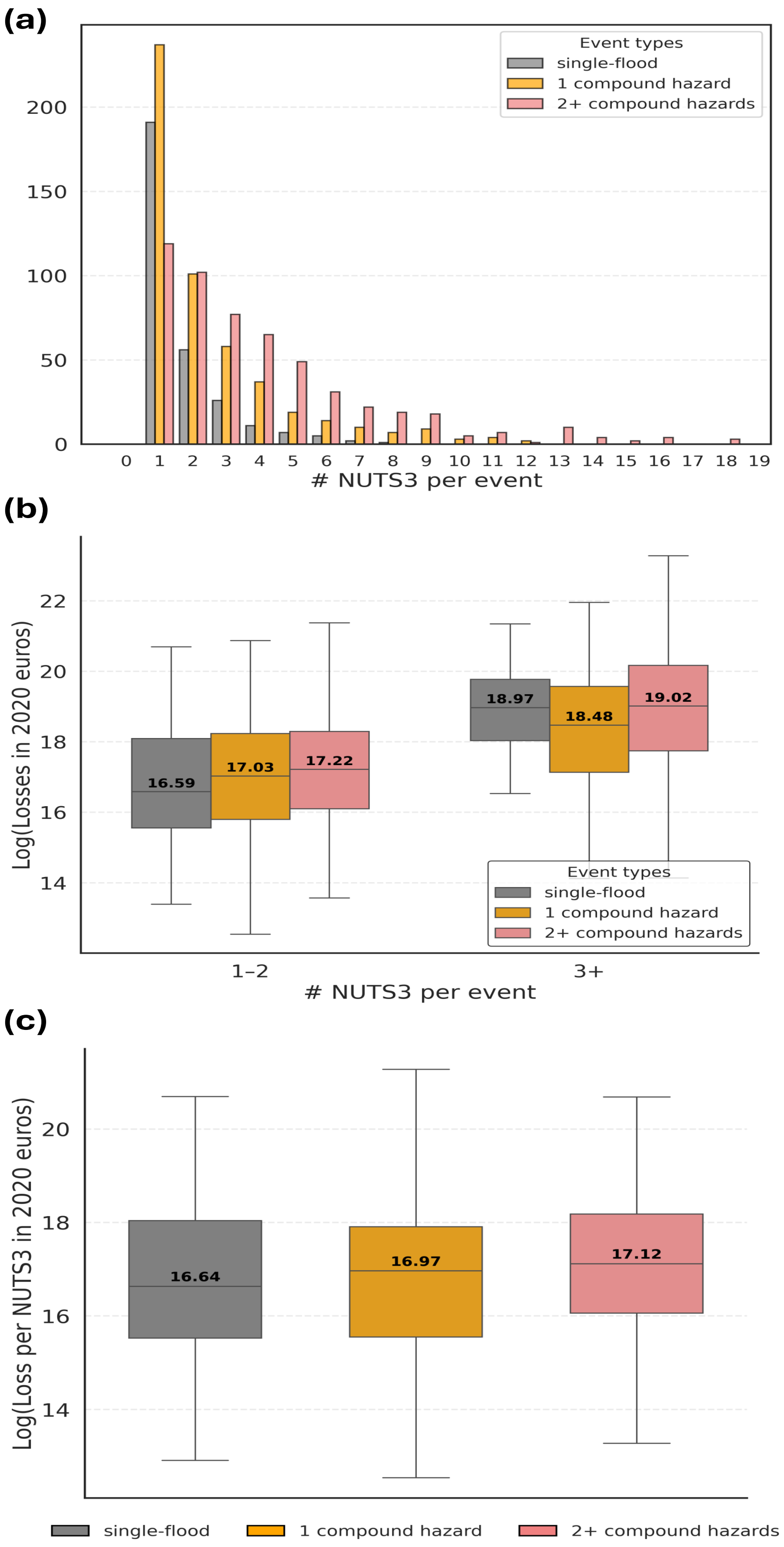

Supplementary Figure 2: Flood footprint size and associated losses. (a) Distribution of flood footprints (number of affected NUTS-3 regions) for single-flood and compound hazard categories. (b) Event-level log-losses stratified by footprint size (1–2 vs. ≥ 3 NUTS-3 regions). (c) Distribution of losses normalized by the number of affected NUTS-3 regions across categories.

Temporal trends in single and compound hazards

To investigate temporal patterns, we analysed the occurrence of single and compound hazard events at the NUTS-3 level across Europe for the years 1981–2020. Single hazards (e.g., heatwaves, windstorms) include all events of that type in a region, irrespective of floods. Compound hazards (e.g., drought–flood) are the subset of single hazards that are spatio-temporally associated with a HANZE flood, using the approach described in Methods. For compound hazards, we tested three temporal windows (14, 28, 56 days) and report results for the literature-informed ("informed") window in the main SI. Events were aggregated at the yearly level for each NUTS-3 region.

Temporal trends were analysed separately for each compound hazard type: when studying a specific pair (e.g., heat–flood), only the primary single hazard (e.g., heatwaves) and the corresponding compound hazard events were included; other hazard types in the same region were ignored for that analysis. Mann–Kendall monotonic trend tests and Sen's slope estimations were applied to assess yearly trends in:

1. All single hazards at NUTS-3 level: droughts, floods, windstorms, heatwaves, coldwaves.
2. All compound hazards at NUTS-3 level: flood–drought, wet sequences, windstorm–flood, heat–flood, cold–flood.
3. The ratio of compound events to the total number of corresponding single-hazard events at each NUTS-3 region.

Results are presented in Figure 3 and Figure 4. Overall, compound hazard types exhibit increasing trends for most pairs, although the magnitude and direction vary across hazard types (Table 4). For drought–flood and heat–flood pairs, both the single-hazard records and the corresponding compound hazards show upward trends, indicating that increases in compound occurrences largely reflect trends in the associated hazard. For wet sequences, single-flood occurrences show no significant trend, while compound hazards increase, suggesting a growing tendency for floods to follow antecedent wet conditions. Windstorm–flood shows no significant trend in either single or compound hazards, though the proportion of windstorms forming compound events increases modestly. Cold–flood pairs display decreasing trends in both single-hazard and compound-hazard records, reflecting the broader decline in coldwave activity. Heat–flood pairs show increasing trends in both single-hazard and compound-hazard records, while the ratio remains relatively stable, reflecting primarily the warming climate. These results indicate that compound hazards largely follow the trajectories of their primary single-hazard drivers, while changes in the proportion of compound occurrences reveal additional shifts in co-occurrence behaviour not always captured by single-hazard trends alone.

**Hazard Type**	**Single Trend (p)**	**Compound Trend (p)**	**Proportion Trend (p)**
Drought	↑ (1.78e-5)	↑ (1.80e-4)	↑ (0.048)
Flood	– (0.41)	↑ (0.044)	↑ (0.0115)
Windstorm	– (0.69)	– (0.093)	↑ (0.018)
Heatwave	↑ (4.64e-12)	↑ (0.013)	– (0.34)
Coldwave	↓ (0.016)	↓ (0.016)	↓ (0.033)

Supplementary Table 4: Mann–Kendall Trends and p-values for Single and Compound Hazard Events at NUTS3 Level (1981–2020). ↑: increasing trend, ↓: decreasing trend, –: no trend.

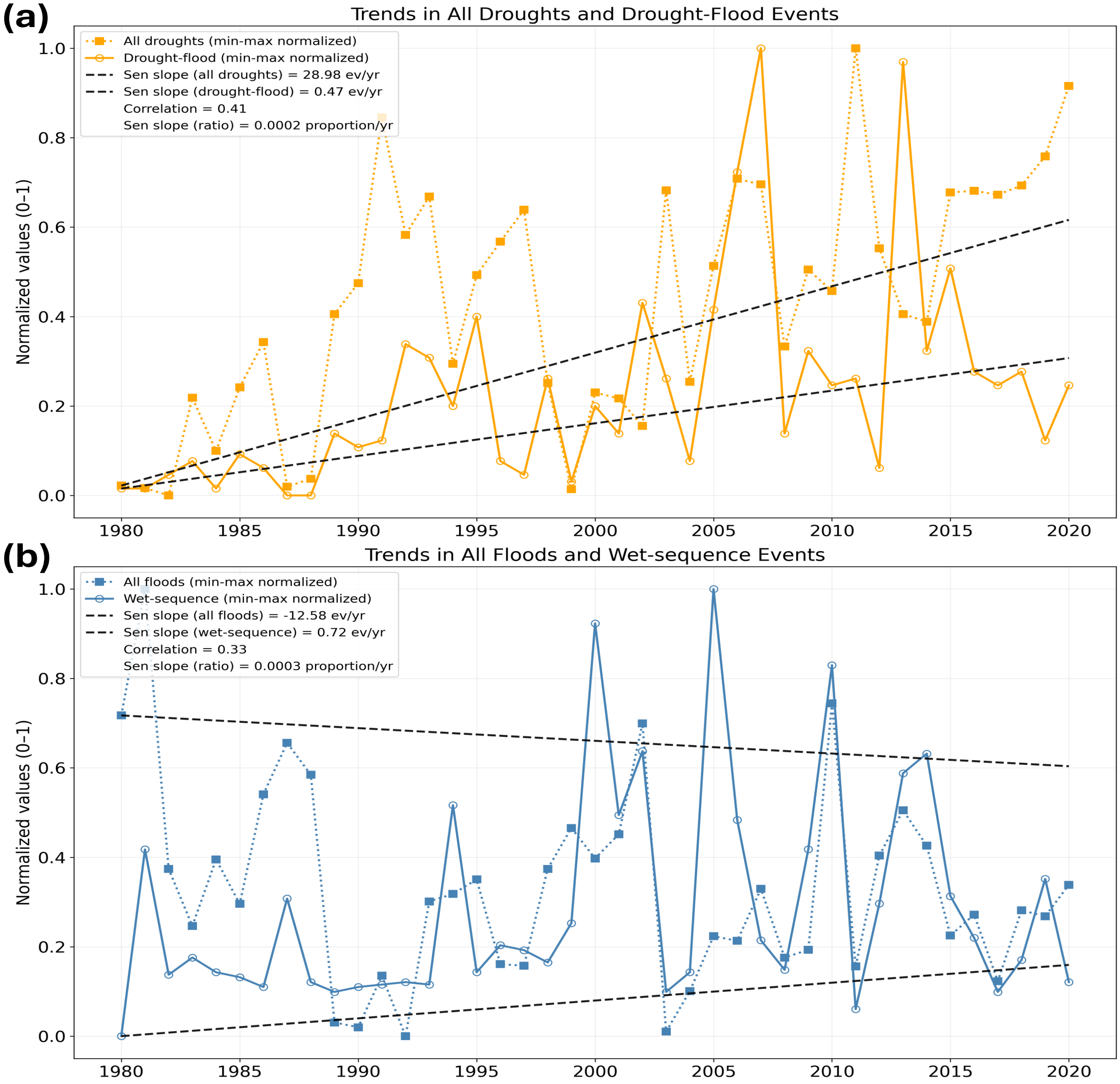

Supplementary Figure 3: Temporal trends of hazard types at NUTS3 level. (a) Normalized annual counts of single drought hazards (dotted) and drought–flood (solid). (b) Normalized annual counts of single flood hazards (dotted) and wet-sequence (solid). Counts are min–max normalized to allow comparison of single vs compound hazards on the same scale. Data are aggregated annually per NUTS3, and trends were quantified using the Mann–Kendall test and Sen's slope.

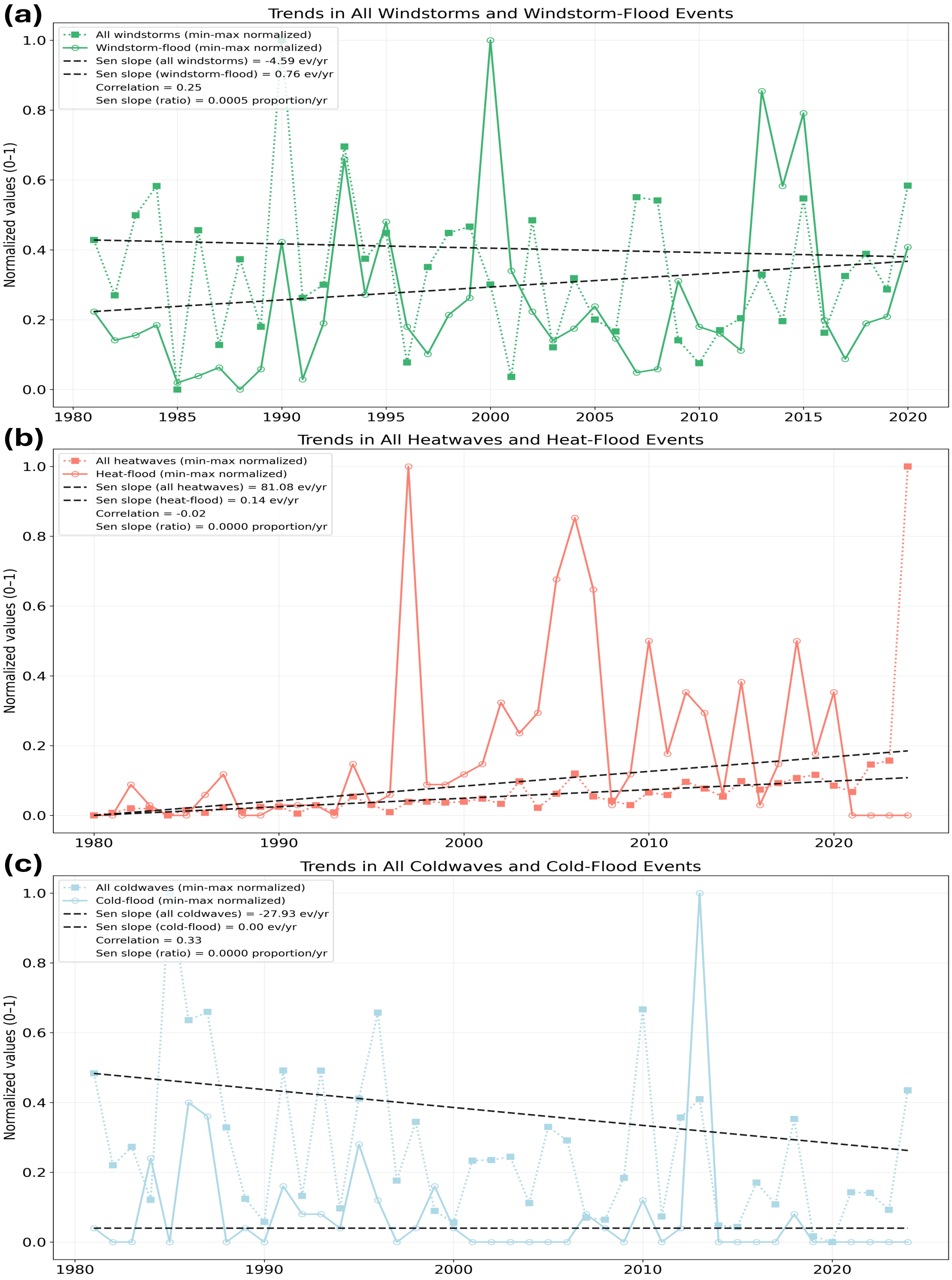

Supplementary Figure 4: Temporal trends of hazard types at NUTS3 level. (a) Normalized annual counts of single wind hazards (dotted) and windstorm–flood (solid). (b) Normalized annual counts of single heat hazards (dotted) and heat-flood (solid). (c) Normalized annual counts of single cold hazards (dotted) and cold-flood (solid). Counts are min–max normalized to allow comparison of single vs compound hazards on the same scale. Data are aggregated annually per NUTS3, and trends were quantified using the Mann–Kendall test and Sen's slope.

Sensitivity of drought–flood compound events to minimum drought duration

To ensure that droughts are hydrologically meaningful and consistent with the 56-day compound-event window, we applied a pooling procedure. Consecutive low-flow episodes separated by less than 30 days were merged into drought clusters, preventing fragmentation of short events and yielding more realistic drought durations. After pooling, we tested four minimum drought-duration thresholds: ≥ 1 day (baseline, all pooled clusters retained), ≥ 7, ≥ 10, and ≥ 14 days. For each threshold, drought–flood compound events were re-identified using the 56-day window, and all floods were re-classified at the event level as either compound drought–flood events or single-flood events. Increasing the minimum drought duration reduces the number of identified drought–flood compounds and the number of unique floods forming compounds, while increasing mean drought length. Supplementary Fig. 5a shows the distribution of event counts across thresholds, illustrating the decrease in compound-event frequency with stricter minimum-duration requirements. Supplementary Fig. 5b presents violin plots of log-transformed flood losses, showing that drought–flood events consistently exhibit higher median losses than single-flood events across all thresholds. Percent differences in mean log-loss between compound and single-flood events range from 1.6% to 2.5%, decreasing slightly as the minimum duration increases. Overall, the results demonstrate that adopting a 10-day minimum drought duration (used in the main analysis) provides a hydrologically meaningful definition while preserving the robustness of flood-loss patterns and compound-event identification. Kolmogorov–Smirnov tests confirm that the log-loss distributions are statistically similar across thresholds, indicating limited sensitivity of flood-impact results to the choice of minimum drought duration.

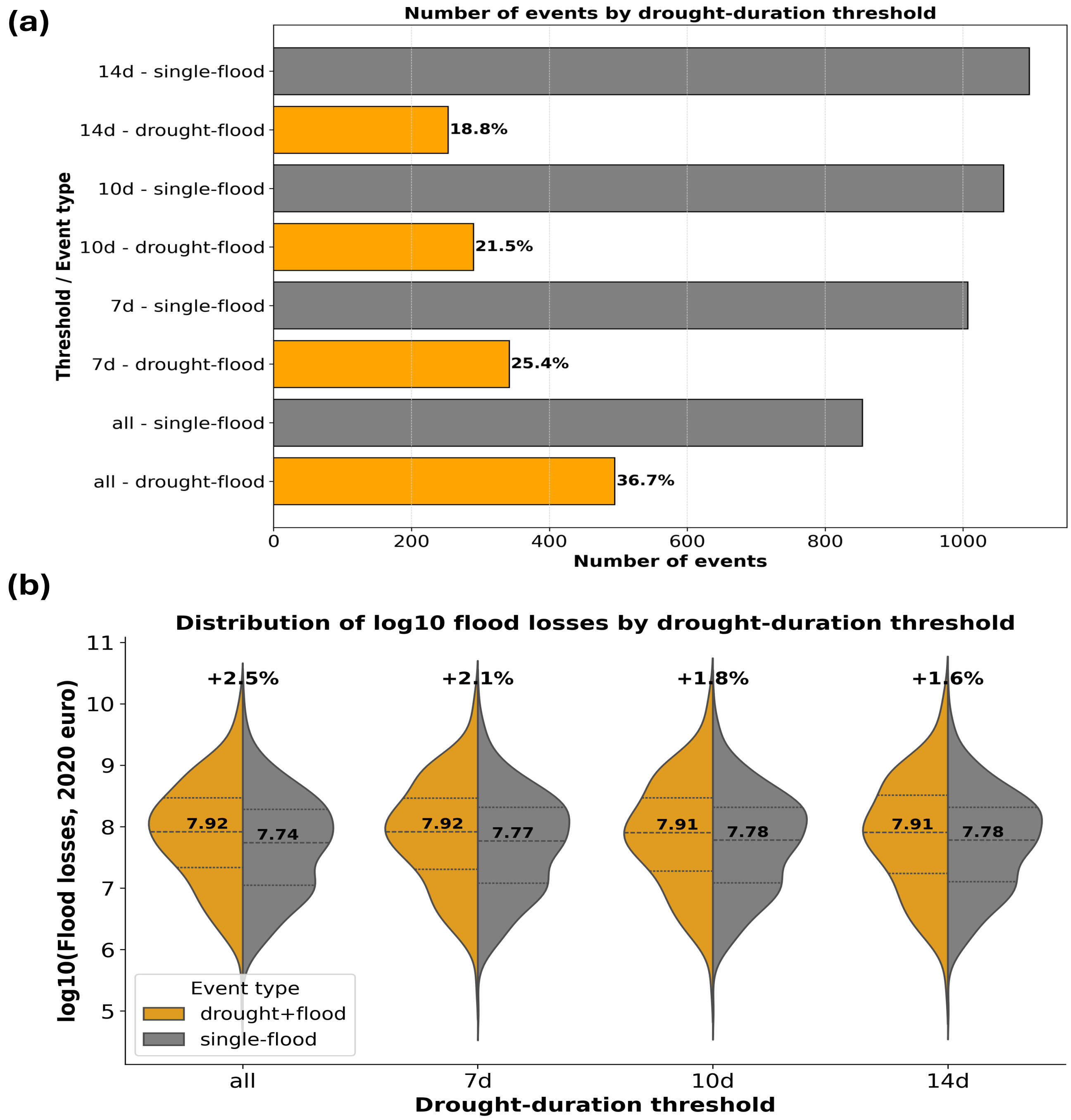

Supplementary Figure 5: Sensitivity of drought–flood compound events to minimum drought duration. (a) Number of events classified as compound drought–flood (orange) or single floods (gray) for four minimum drought-duration thresholds (≥ 1, 7, 10, 14 days). Increasing the minimum duration reduces the number of compound events and increases mean drought length. (b) Distribution of log flood losses for compound drought–flood (orange) and single-flood (gray) events across the same thresholds. Median losses for compound events are consistently higher, while Kolmogorov–Smirnov tests indicate that the overall loss distributions remain statistically similar across thresholds. Percent differences in mean log-loss between compound and single-flood events are shown above the violins.

Regional distribution

At the subnational scale, our dataset includes all HANZE flood records mapped to the NUTS-3 administrative regions of Europe, together with their compound-hazard classifications derived from region-level matching. When a HANZE flood event affects multiple NUTS-3 regions, the reported economic loss is divided equally among the affected regions to maintain consistency between the spatial disaggregation of events and the regional loss totals. Each affected NUTS-3 region is evaluated independently for co-occurring drought, heat, cold, wind, or additional flood anomalies, and an event is classified as compound when at least one affected region exhibits a concurrent hazard within the defined temporal window. The total occurrences at NUTS3 of the five hazards under consideration are shown in Figure 6 (droughts in (a) and floods in (b)), Figure 7 (heatwaves in (a) and coldwaves in (b)), and Figure 8 for windstorms. Figures 9 and 10 summarise the resulting spatial patterns when aggregated over the full study period. The first pair of maps shows the total number of floods recorded per region and the subset identified as compound events, revealing distinct spatial concentrations in both overall flood occurrence and compounding. The second pair displays cumulative economic losses after regional allocation, alongside the spatial distribution of compound-hazard complexity. Together, these panels provide a regional overview of flood occurrence, associated losses, and compound event interactions across Europe, forming the subnational basis for the event-level analyses presented in the main text.

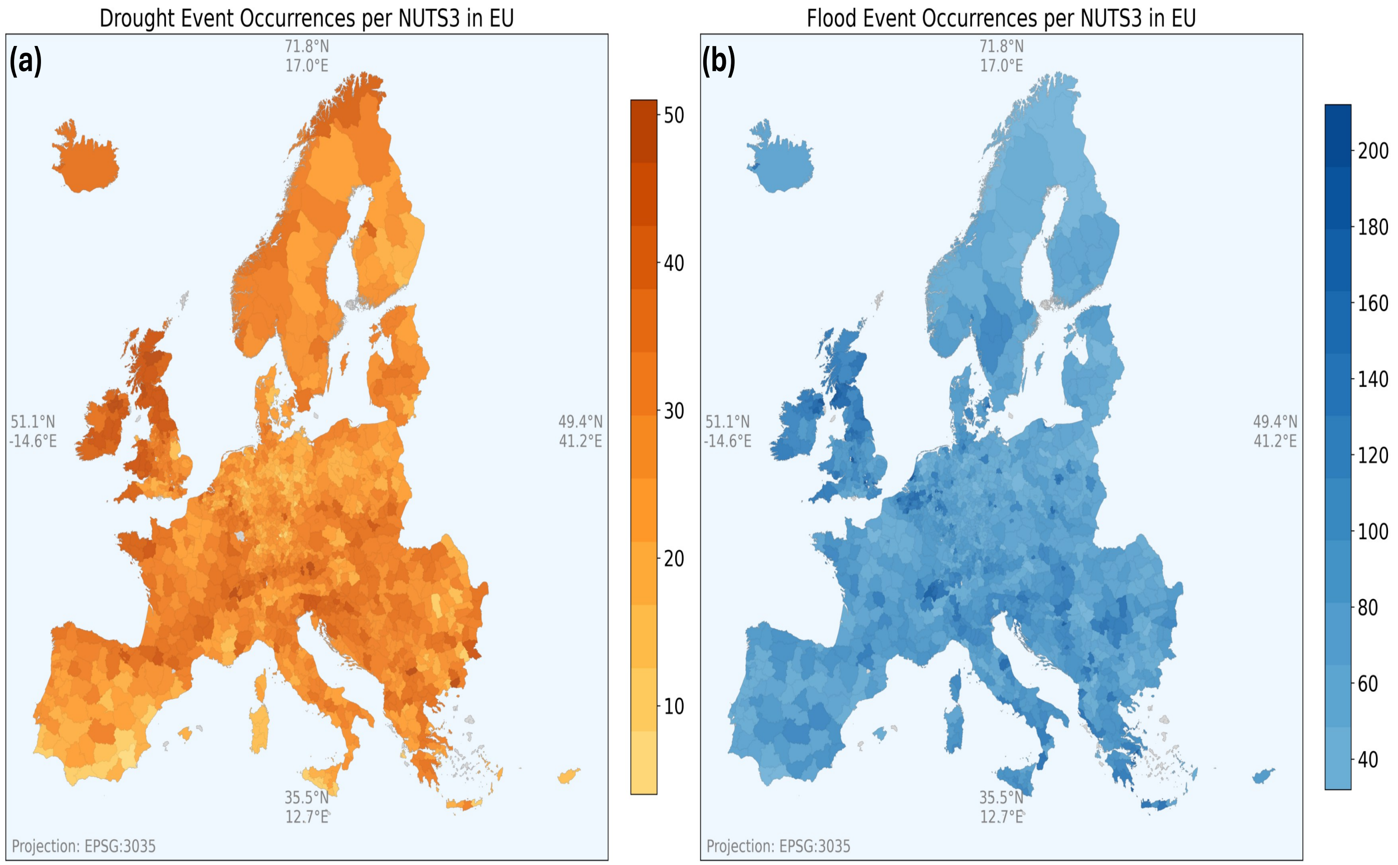

Supplementary Figure 6: Distribution of hazards per NUTS3 during the study period from 1981 to 2020. (a) Number of droughts recorded per region and (b) number of floods recorded per region.

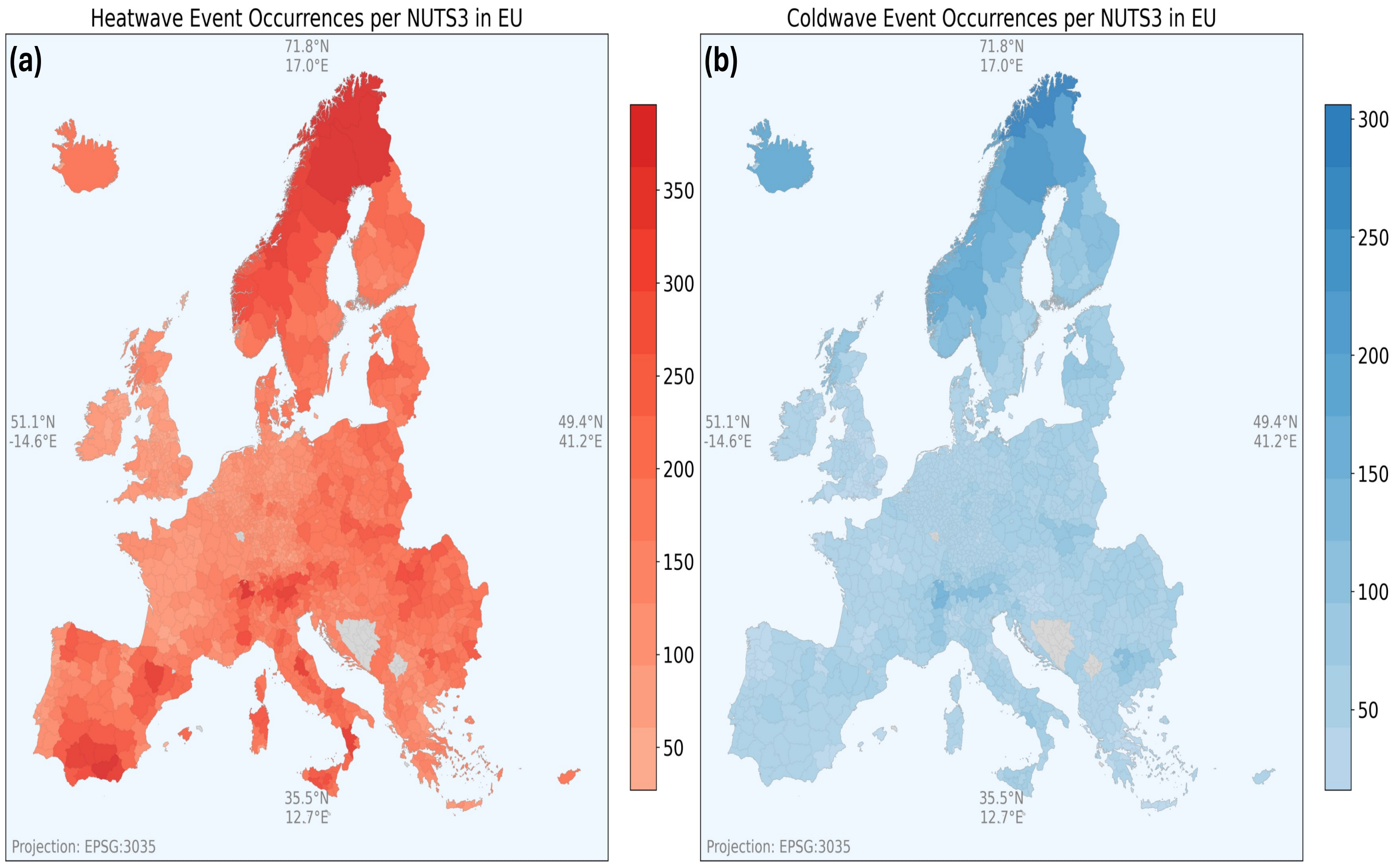

Supplementary Figure 7: Distribution of hazards per NUTS3 during the study period from 1981 to 2020. (a) Number of heatwaves recorded per region and (b) number of coldwaves recorded per region.

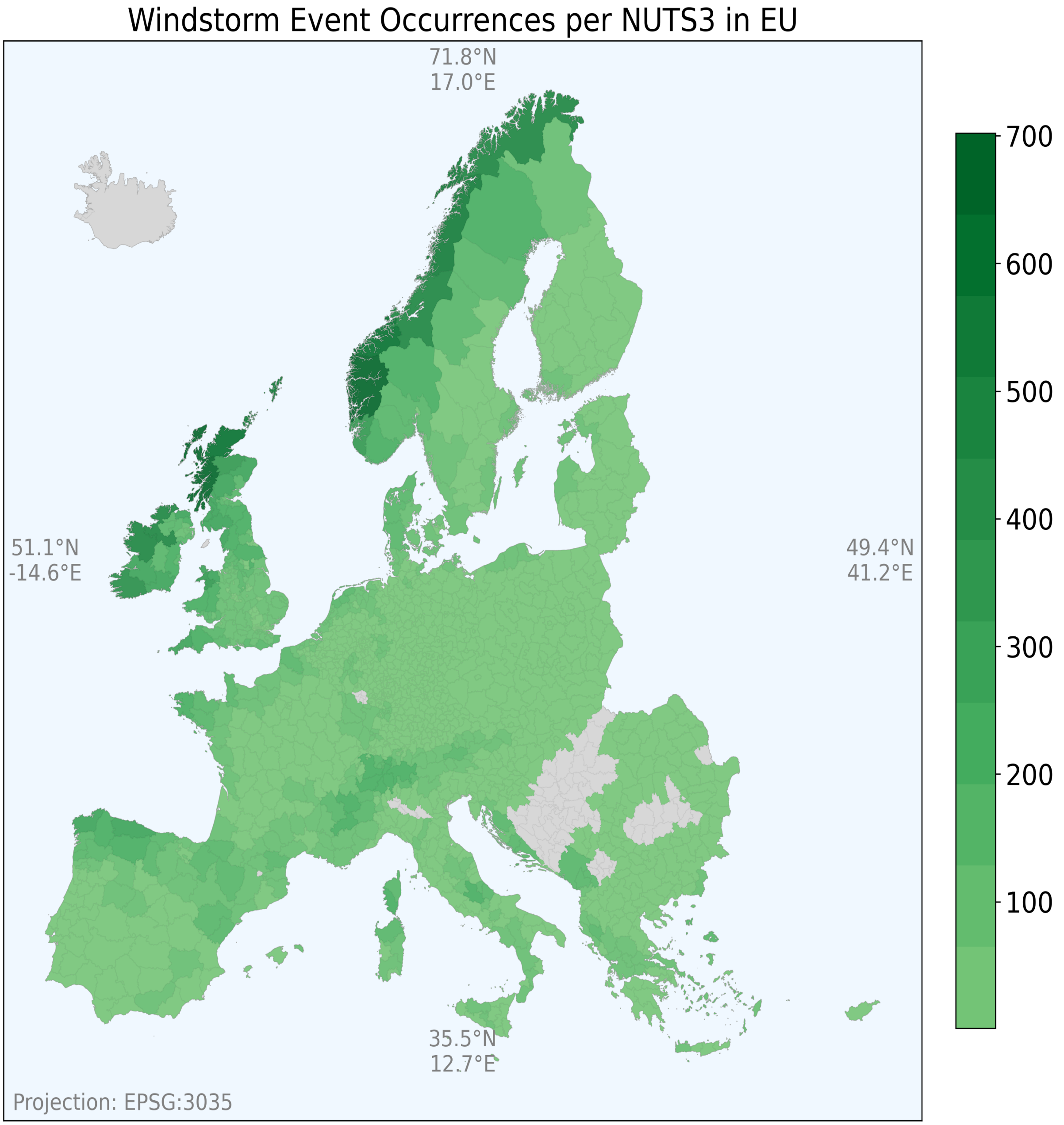

Supplementary Figure 8: Distribution of windstorms across NUTS-3 regions (1981–2020). Spatial distribution of windstorm occurrences aggregated at the NUTS-3 level over the study period. The map highlights regional variability in windstorm frequency, with higher concentrations indicating areas more frequently affected by windstorm events.

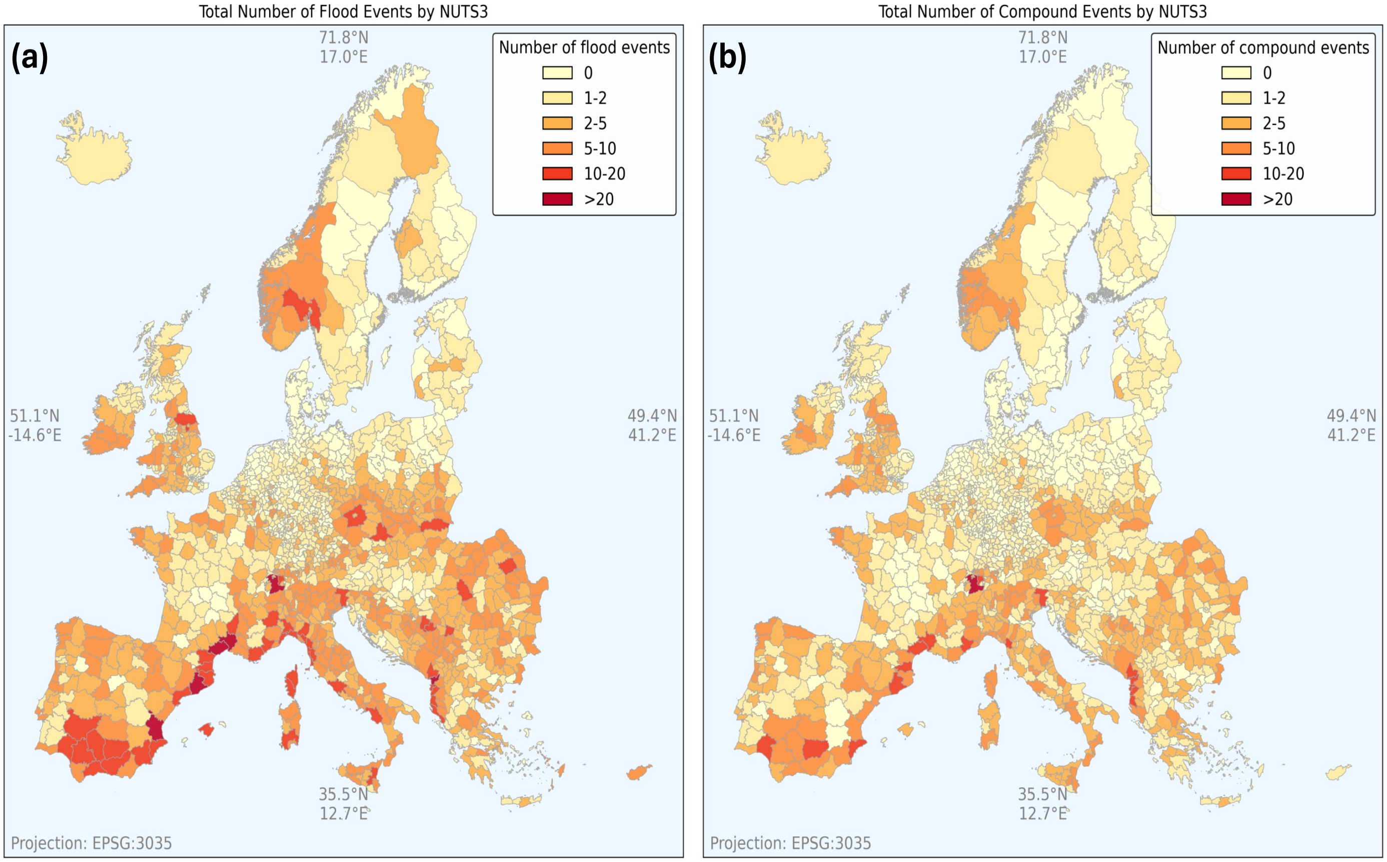

Supplementary Figure 9: Distribution of all floods and compound events across Europe at the NUTS-3 level. a) Number of all floods recorded per region and (b) number of compound events recorded per region.

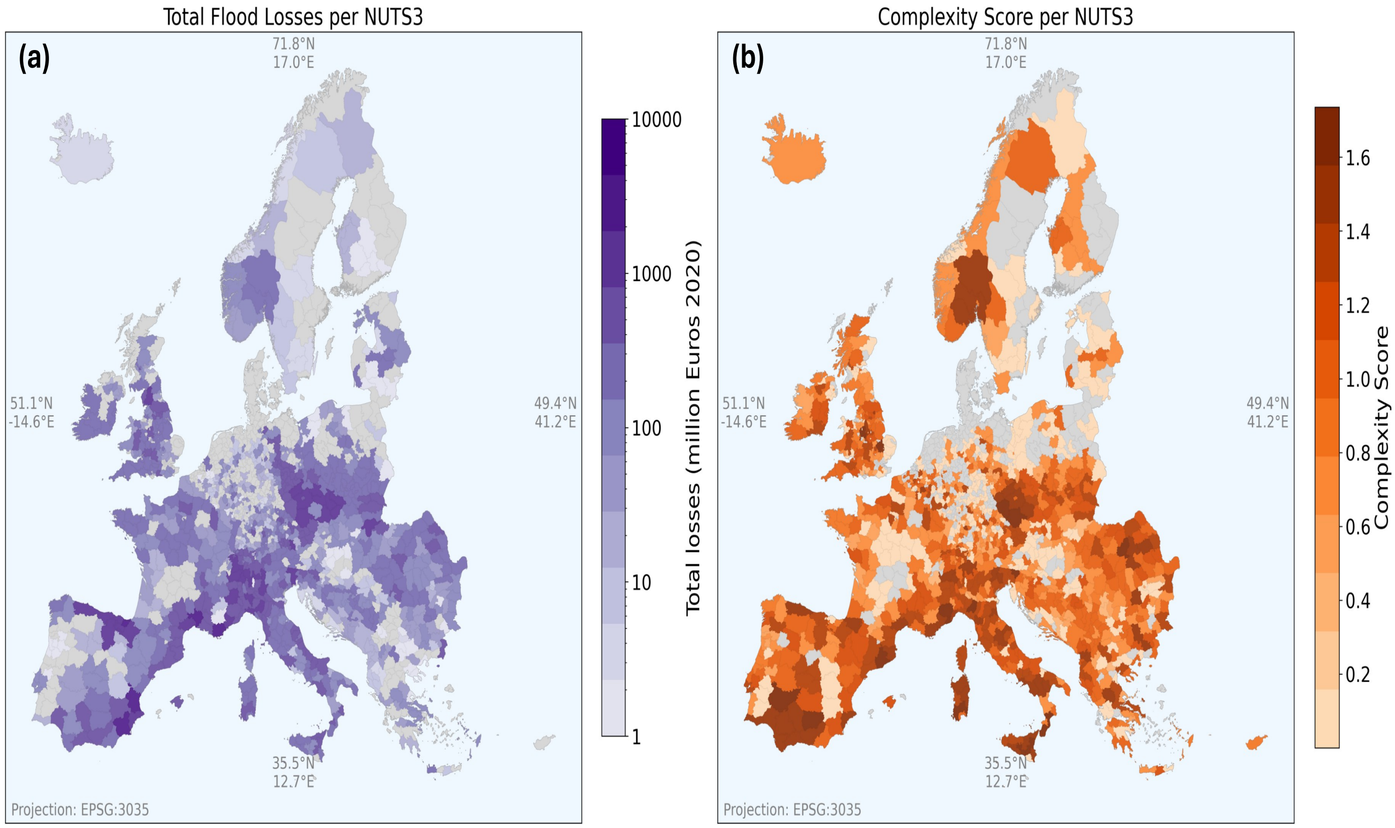

Supplementary Figure 10: Comparison of flood impacts and compound hazard complexity across NUTS3 regions in Europe. (a) Total flood losses per NUTS3 region (million euros, adjusted to 2020 values). Regions with no reported losses are shown in light grey. (b) Compound hazard complexity per NUTS3 region.

Training with bootstrapping

To quantify predictive performance and assess model robustness, we trained GBM regressors on 300 bootstrap-style random splits, using 90% of NUTS3 observations for training and 10% for testing. Each split sampled from the full predictor set—which includes compound-hazard indicators (pre-hazard counts, lagged timing, intensities, and the compound hazard complexity index), exposure metrics (population and built-up area), flood magnitude (return period), and socio-economic vulnerability indicators (RDH-VI, SoVI, Gini, GDP, HDI). The correlation structure among these predictors and the target variable is shown in Fig. 11, highlighting both expected associations and cases of moderate multicollinearity that the GBM framework can accommodate through regularization. Hyperparameters were tuned through randomized search with constraints that act as structural regularization: shallow trees (maximum depth 2–4), relatively large min samples split and min samples leaf, stochastic subsampling of rows and columns, and a learning-rate penalty on successive boosting iterations. These restrictions limit model capacity and reduce the risk of overfitting.

Model performance across the 300 resamples is summarised in Fig. 12. Training R^2 values average 0.47, while test R^2 averages 0.22. The mean train/test MSE ratio is approximately 0.7, indicating that the generalisation gap remains moderate and performance does not deteriorate sharply on hold-out samples. We therefore acknowledge some degree of overfitting, but note that both the structural regularisation and repeated resampling provide a conservative estimate of generalisation error. Importantly, SHAP values are computed on test sets for each split and then averaged across all 300 repetitions (see Fig. 13a-c), yielding stable, well-converged estimates of feature contributions and interactions that are robust to sampling variability. Permutation importance (Fig. 13d) was used as an additional diagnostic to verify that dominant predictors are consistent across splits and regularisation settings. Together, these steps provide a comprehensive assessment of model behaviour, mitigate overfitting risk, and support the reliability of the feature-level insights reported in the main text.

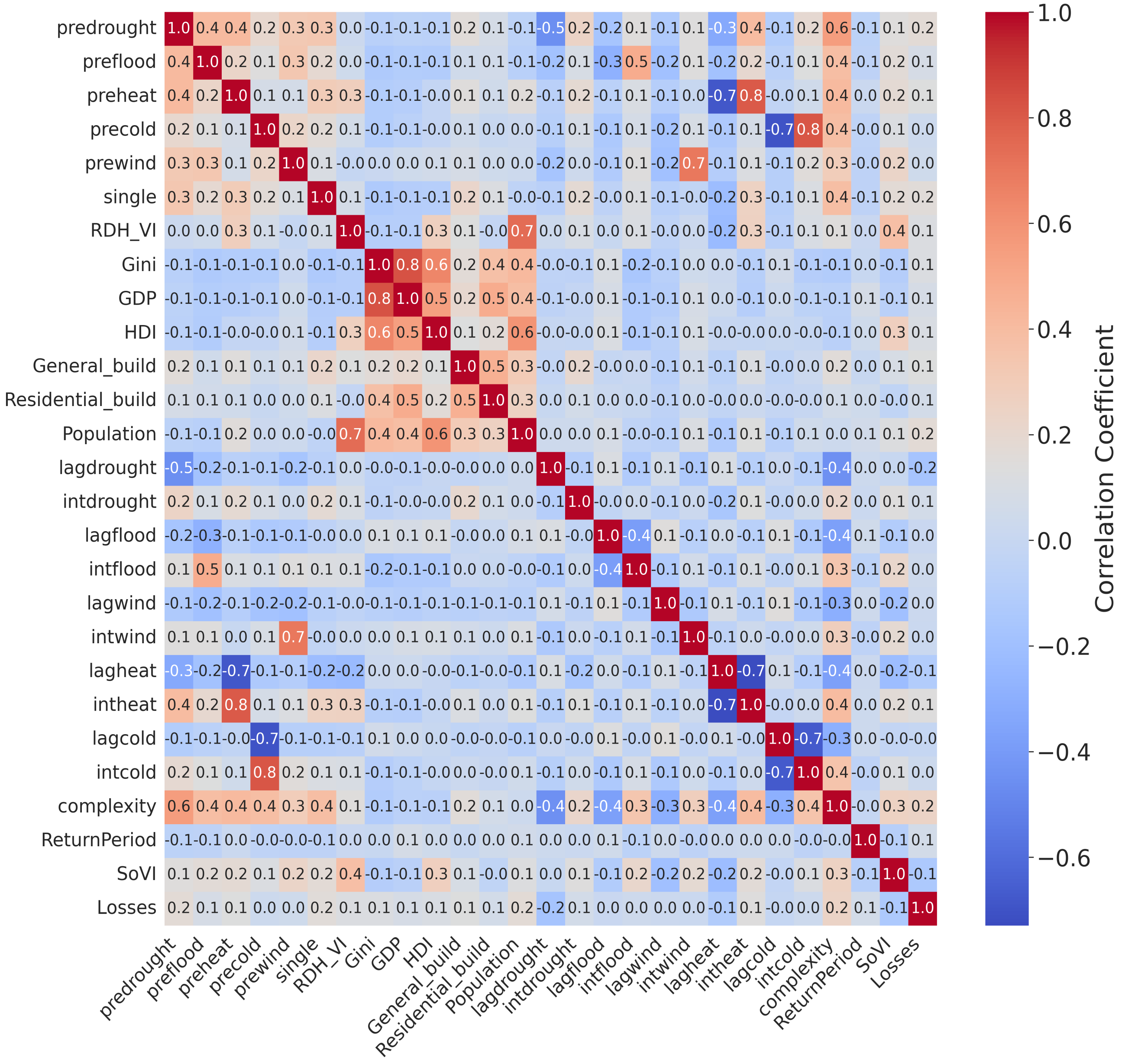

Supplementary Figure 11: This matrix illustrates correlation coefficients among all predictors and the target variable, log mean Loss. Positive correlations are indicated by red hues, while negative correlations are shown in blue. The color intensity reflects the correlation strength, highlighting key relationships and potential multicollinearity.

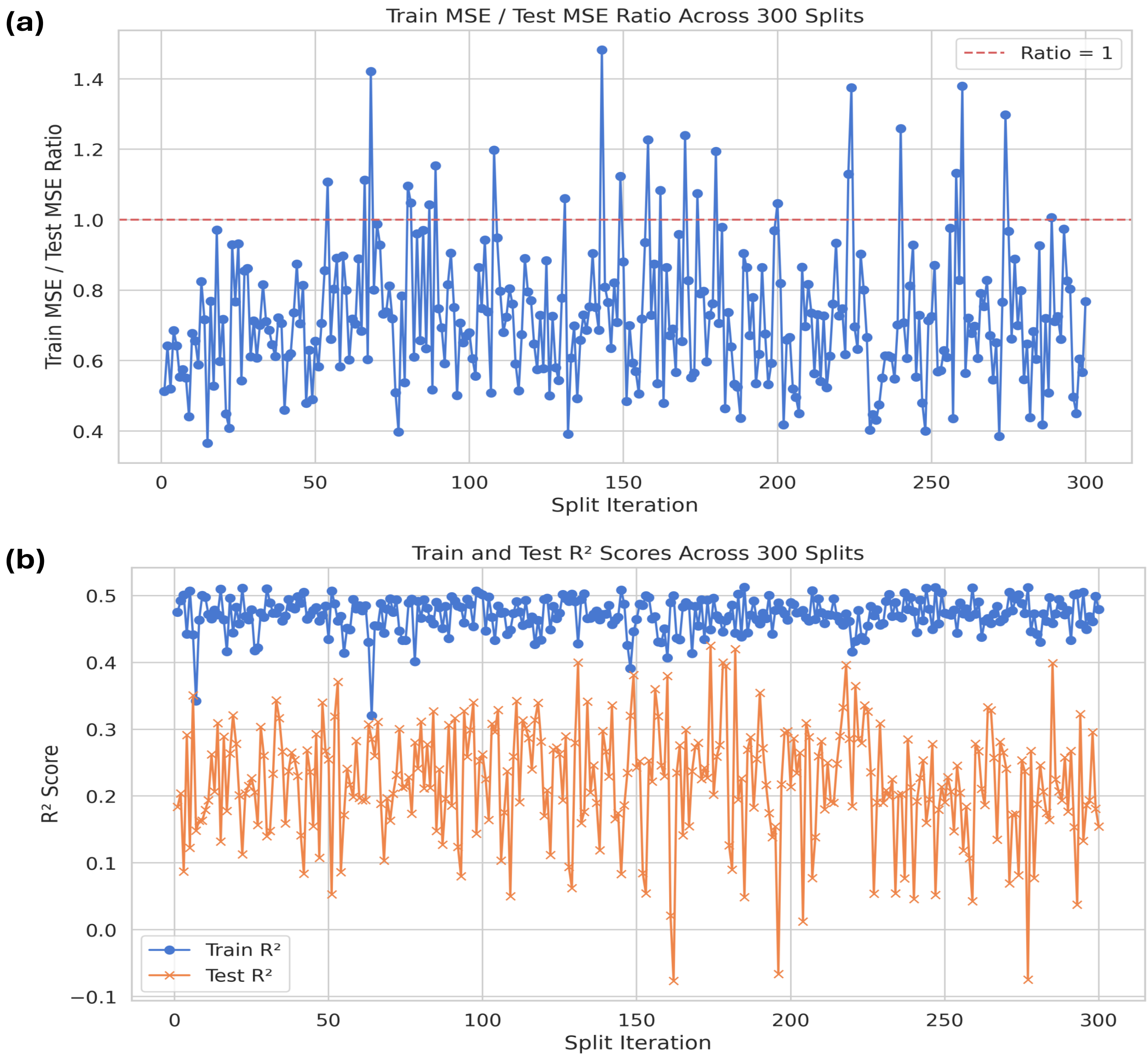

Supplementary Figure 12: Summary of GBM performance across 300 random 90/10 train-test splits. (a) The ratio of Mean Squared Error (MSE) for train and test sets, highlighting a degree of overfitting as indicated by ratios below 1. (b) R^2 values for train and test sets, showing that while test R^2 is noticeably lower than train R^2 , the model still demonstrates predictive skill.

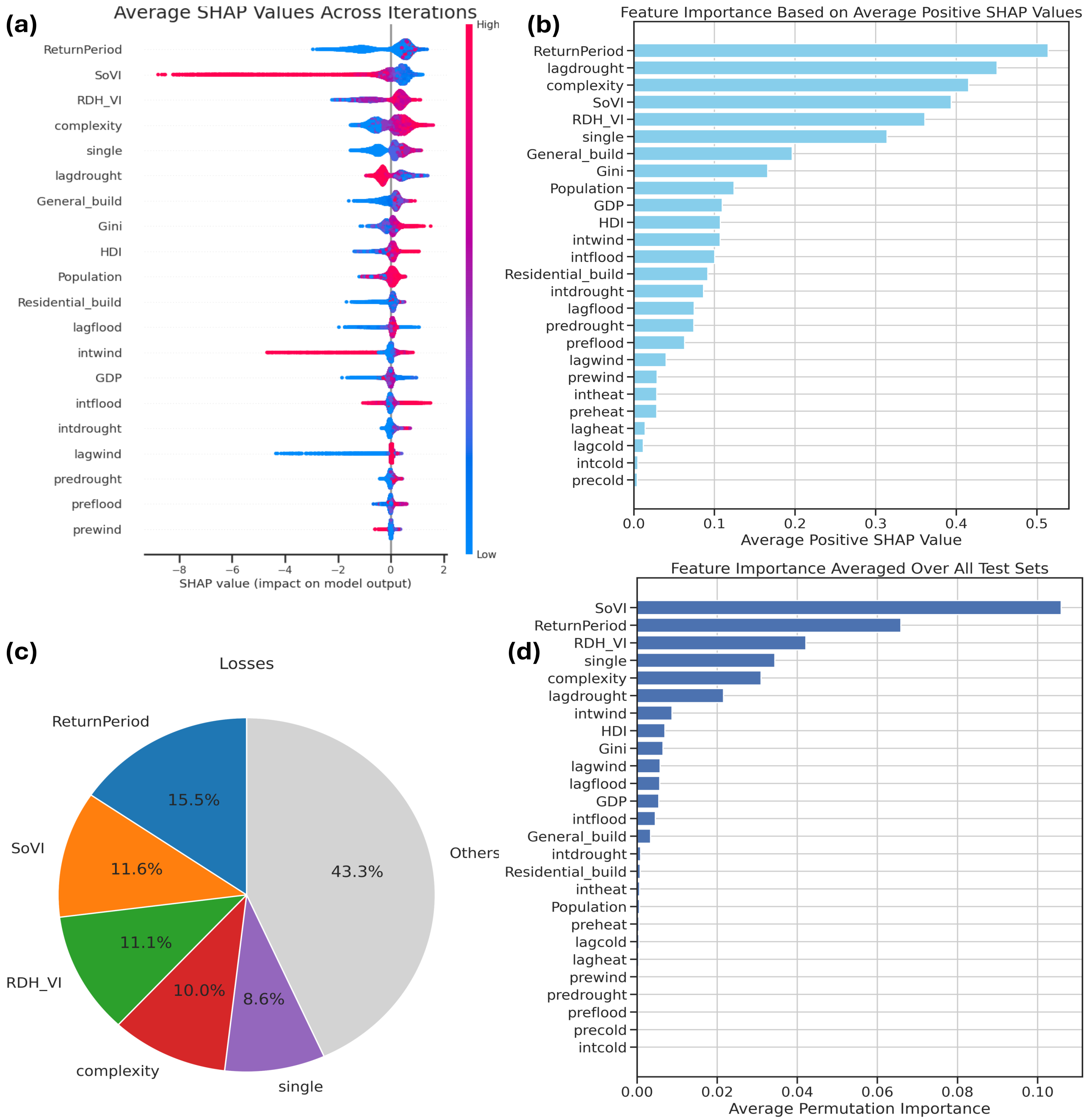

Supplementary Figure 13: Explainable AI analysis from ensemble GBM models trained on 300 random 90/10 data splits. The xAI results are derived from test sets, with explanations averaged across all trained models to ensure robustness. (a) A SHAP beeswarm plot depicts the influence of features on model predictions, with feature colors representing normalized feature values. (b) The average positive SHAP values are presented, highlighting features that contribute positively to increasing the average log losses predicted by the GBM models. (c) A pie chart illustrates the relative contribution of the top five features, expressed as a percentage, based on the average absolute SHAP values. (d) Feature importance is assessed using permutation importance, offering a complementary perspective on how feature perturbations affect model performance.

Interactions among drivers

These analyses, shown in Figure 14, explore how hazard complexity, flood magnitude, and vulnerability relate to economic losses. Panel (a) shows mean log-transformed losses for high- and low-complexity regions within quartiles of the average return period (Low, Med-Low, Med-High, High). Across all quartiles, high complexity samples (i.e., with values exceeding the 75th percentile) consistently exhibit higher losses ($p < 0.03$ for all quartiles), with p-values computed using two-sample t-tests assuming unequal variances (Welch's t-test) between high- and low-complexity events within each quartile. This indicates that hazard complexity increases impacts even when flood magnitude is comparable. Panel (b) presents the distribution of SHAP values for complexity across the same standardized quartiles, split by vulnerability (RDH-VI: Low vs High). Complexity SHAP values are generally higher for high vulnerability, with the difference being most pronounced in the lower magnitude quartiles, while medians are closer in the higher quartiles. These patterns suggest that while there may be some interactions with return period and vulnerability, their magnitude is moderate, and hazard complexity primarily exerts an independent effect on losses. Together, these panels provide complementary descriptive evidence supporting the conclusion from the causal analysis that complexity directly amplifies economic impacts, with only modest mediation through return period or vulnerability.

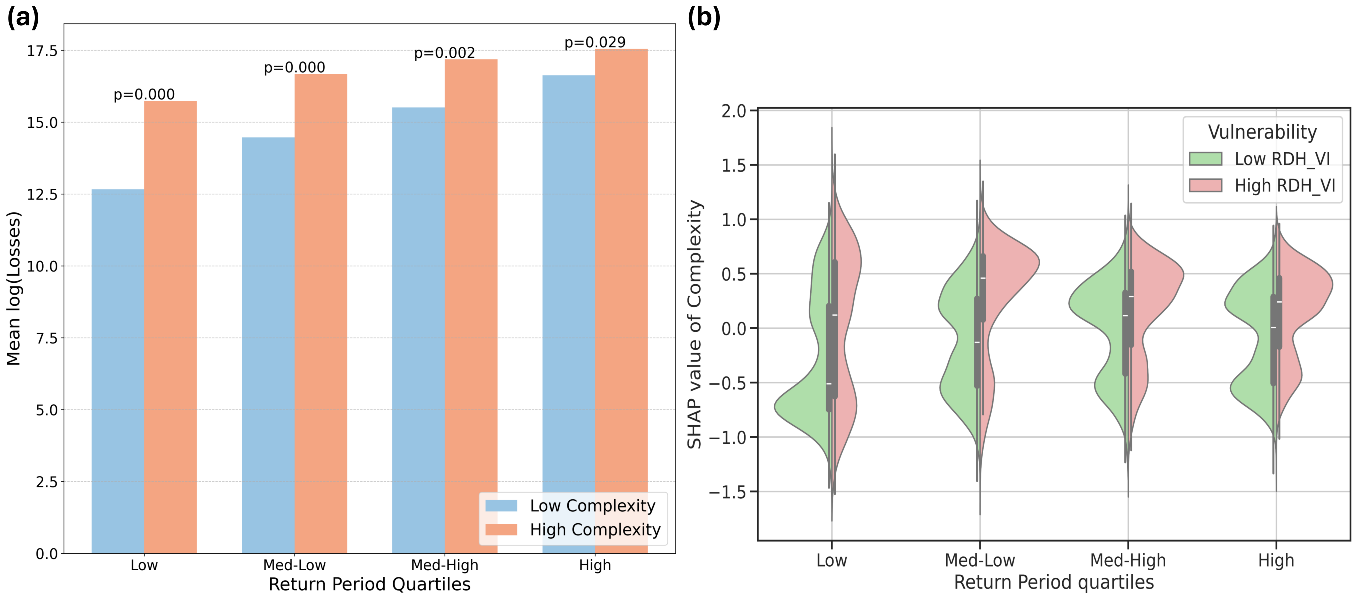

Supplementary Figure 14: Hazard complexity, flood magnitude, and economic losses. (a) Mean log-transformed losses for high- and low-complexity events within return period quartiles (Low, Med-Low, Med-High, High). High-complexity events consistently exhibit greater losses across all quartiles, indicating that complexity increases impacts even for comparable flood magnitude levels. (b) Distribution of SHAP values for complexity across standardized return period quartiles, split by vulnerability (RDH-VI: Low vs High). This panel illustrates both the direct effect of complexity and its interaction with return pediod and vulnerability in the predictive model.

Modelling Fatalities and People Affected

In this section, we employed a bootstrapped GBM approach to model log-transformed fatalities and people affected, leveraging explainability techniques to characterise model behaviour. The model's performance, summarised in Table 5, indicates that the average explained variance is comparable for both targets, with test values slightly higher than those obtained for losses discussed in the main text. Figure 15 provides detailed insights into feature importance and directionality using both SHAP values and permutation importance. Panels (a) and (c) show SHAP beeswarm plots, where feature values are depicted by colour gradients, while panels (b) and (d) report permutation importance, quantifying the decrease in model performance when each predictor is randomly permuted. Panels (a)–(b) refer to fatalities, and panels (c)–(d) to people affected. Notably, vulnerability indicators—particularly SoVI—play a central role in modelling fatalities, reflecting its design to capture cumulative flood-related mortality. In contrast, compound-hazard complexity emerges as the dominant factor for predicting people affected, consistently highlighted across both SHAP and permutation results. These findings align with the feature-importance patterns observed for losses, reinforcing expected relationships across different impact metrics.

**Target**	**Train** R^2	**Test** R^2
Fatalities	0.53	0.32
People Affected	0.53	0.33

Supplementary Table 5: Model Performance Metrics

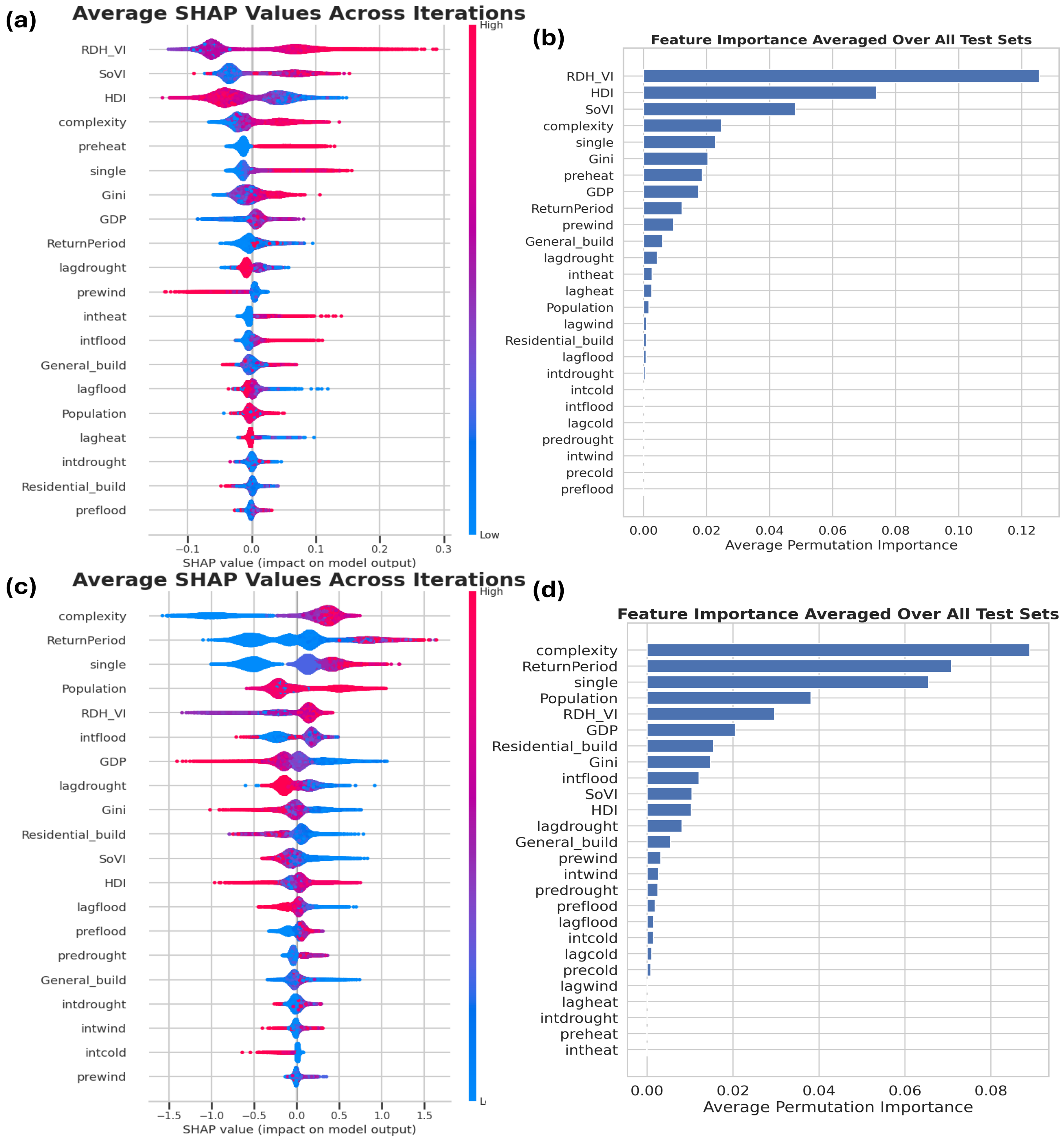

Supplementary Figure 15: Modelling (log) Fatalities (panels (a) and (b)) and (log) Affected People (panels (c) and (d)). (a)-(c) A SHAP beeswarm plot depicts the influence of features on model predictions, with feature colors representing normalized feature values. (b)-(d) Feature importance is assessed using permutation importance, offering a complementary perspective on how feature perturbations affect model performance.
