# Supplementary material for: Compounding hazards increase flood economic losses across Europe
Source: Nat Commun. 2026 May 19;17:6614. doi: 10.1038/s41467-026-73248-0 (PMC13381573; doi:10.1038/s41467-026-73248-0)
Supplement: Supplementary file 2 — Peer Review file [file 41467_2026_73248_MOESM2_ESM.pdf]

nature portfolio

Peer Review File

Compounding hazards increase flood economic losses across Europe

Corresponding Author: Dr Michele Ronco

This file contains all reviewer reports in order by version, followed by all author rebuttals in order by version.

Version 0:

Reviewer comments:

Reviewer #1

(Remarks to the Author)
This manuscript compiles and analyses a European-wide dataset of flood impacts (economic losses) associated with compounding hazards. In my view, its most significant contributions are the dataset itself (including the proposed new metric) and the finding that all the costliest events were linked to multiple hazard types. The examination of how vulnerability and exposure influence the relationship between hazard complexity and losses is also valuable, but weaker in execution in its current form.

I welcome this initiative and agree that combining existing impact data with hazard, exposure, and vulnerability information is a promising way to maximise the value of available datasets. That said, the manuscript requires substantial revisions before it can be considered for publication in Nature Communications. Below, I outline my major concerns and suggestions for improvement. This study has strong potential, and addressing the points raised would substantially enhance its clarity, methodological soundness, and interpretive depth.

1. Interpretation of main findings
The manuscript should more clearly acknowledge that feature importance from SHAP analysis does not equate to causality. Greater discussion is also needed on the relationship between hazard complexity and losses. Is it the hazard interactions or the vulnerability interactions that exacerbate impacts? While Table 1 offers a theoretical overview, the dataset could potentially be used to empirically identify which interactions tend to amplify economic losses. The claim that unpredictability drives losses (Results, paragraph 4) would benefit from supporting analysis. In essence, does hazard complexity increase impacts for the same flood severity, or does it instead amplify flood severity, indirectly increasing losses? Controlling for flood severity in the analysis could help clarify this, still open, question. And considering that the model only shows moderate performance, how does this affect your results?

2. Event duplication and definition
Duplicating records when an event is associated with more than one compounding hazard type is problematic, as it may bias results. A more robust solution could, for instance, be to introduce an additional archetype for these events. Relatedly, the definition of an “event” is unclear. Is it a single HANZE record? Could multiple records represent the same physical event across administrative regions, and is this accounted for in the analysis? Given that the model likely assumes independence between observations, correlated records from the same event could bias outcomes. This should at least be acknowledged, if not addressed analytically.

3. Sample transparency
The manuscript should report the total HANZE sample size, and the number identified as compound events. The figure of 1,955 compound events is given, but without reference to the total number of records or the extent of duplication.

4. HANZE database description
The description of the HANZE database should be more comprehensive, for instance clarifying data sources, included flood types, disaster inclusion criteria, and potential spatiotemporal reporting biases. The rationale for including only riverine and flash floods should be explained, and whether HANZE’s “compound” category of river + coastal is included. The large number of records with “no recorded impacts” also need clarification; do these represent true zero-loss events or missing data?

5. Archetype context and hazard representation
The archetypes are well explained, but their brief descriptions might be moved from the Methods to the Introduction for early context. In the Emilia-Romagna example, consider briefly explaining how drought preconditions exacerbated the flood impacts. My main question related to the archetypes is why you do not analyse precipitation as a form of wet sequence?

6. Focus and scope
The particular focus on flood impacts should be highlighted earlier in the Introduction. The paper opens with a general discussion on multi-hazards, but I think the transition to focusing only on flood impacts could be written in a more clear and justified manner. And more importantly, regarding impact classes, the rationale for excluding other impact metrics available in HANZE (e.g., fatalities, affected people) needs to be better justified.

7. Vulnerability and exposure variables
The rationale for linking social vulnerability data to economic losses needs more theoretical grounding. Economic losses often correlate with asset value rather than social vulnerability. You might want to consider choosing only the vulnerability indicators most relevant to economic damages since this is what you focus on. Moreover:
• Which variables do you mean represent infrastructure vulnerability (mentioned in discussion, first paragraph)?
• The manuscript should also acknowledge and discuss potential collinearity among vulnerability and exposure predictors, and how this might affect the results.
• If kept in analysis, I think that more detail is needed on the vulnerability datasets, including a more thorough reporting of the factors behind the RDH VI index. And is the global SoVI applicable to a European context?
• Regarding exposure, choice of built-up area as the sole exposure metric should be explained, why not population data?
• Regarding data aggregation, why use average exposure and vulnerability values over the study period when dynamic data are available? And the methodology for spatial aggregation is unclear. While averaging across NUTS regions may be appropriate for some variables, its meaning is less obvious for datasets such as built-up area. How should the resulting averaged values be interpreted?

8. Trends and statistics
When identifying trends, specify whether statistical tests were performed. Clarify whether trends in drought or heat compound events mirror trends in individual hazards or reflect a growing compound nature.
The rationale for using average flood damages (rather than another statistic) should be discussed.
The use of flood return periods should also be more clearly described, including data coverage and calculation method.

9. Terminology and clarity
Across the manuscript, the terms “events”, “archetypes”, and “hazard types” appear to be used somewhat arbitrary and interchangeably. For example, in reference to the “event complexity” metric, described as measuring “how varied the mix of events is”, but the measure is really the mix of hazard types, right? This distinction should be clarified and referred to in a consistent manner throughout the manuscript.

More Detailed Comments
• Abstract: The phrase “the Early Warnings for All initiative” may not be familiar to all readers. A brief explanation would be helpful.
• Introduction (first sentence): The term “driver” may not be clear to all readers. Consider clarifying.
• Table 1: The table caption would benefit from explaining what the numerical values represent, rather than repeating the list of archetypes (which are already shown in the table). The numbers themselves could instead be explained in the Methods section. Also, the in-text listing of archetypes is not fully consistent with Table 1, consider aligning these for clarity.
• Figure 1: Consider using the colour white to represent “zero” consistently across all panels. Additionally, it might be more informative to also visualize the accumulated frequency of events and economic losses across the administrative regions.
• Figure 2: Panel a: The text above the pie charts is too small to read; clarify what one dot represents and define “MH events.” Panel c (should be b): Clarify what the dots with connected lines represent. Panel b (should be c): Clarify how 10-year averages were calculated at the start and end of the analysis period.
• Results, paragraph 3: The phrase “We group newly created events” is unclear—what is meant by “newly created”? Also, providing information on how the losses (e.g., category intervals) differ between impact classes would be informative.
• Results, paragraph 4: Use a single, consistent term for the new metric rather than multiple names.
• Results, paragraph 5: Specify the modelling approach more clearly, “supervised learning framework” is vague.
• Novel metric: The description of the novel metric should also appear in the Methods section for completeness.
• Supplementary Information, Figure 5: Clarify the meaning of “chosen for enhanced visual clarity.”

(Remarks on code availability)

Reviewer #2

(Remarks to the Author)
I have reviewed the paper “Compounding hazards increase flood economic losses across Europe” by Ronco et al. In this paper, the authors analyze an existing database of recorded floods and flood impacts across Europe over 1981-2020. For each recorded flood, the authors analyze atmospheric reanalysis data, hydrologic reanalysis data, and/or observations to determine whether the flood occurred simultaneously or following extreme heat, extreme cold, drought, extreme wind, or wet antecedent conditions. Floods associated with one of these other events are classified as compound events.

To analyze flood impacts associated with compound events, the authors first define a “compound event complexity” for each EU NUTS-3 region, which is the cross-entropy of the number of compound events of each type in that region. The compound event complexity is then used as a predictor in a Gradient Boosting Regression machine learning model to predict average flood losses for each region. Other predictors to the model include average flood intensity and metrics summarizing exposure and vulnerability in each region. Using Shapley values (an explainable AI method to analyze the influence of different predictor variables), the authors claim that their event complexity metric is the most important driver of higher average flood losses in a region. Ultimately, this leads to statements that

While this paper explores an important topic of how floods (including compound flood hazards) affect flood losses, my assessment is that the methodology is not sufficient to support the types of causal claims that the authors make in the text. I have outlined some of these main concerns below. Overall, I would not consider this paper currently suitable for publication.

Main Comments:

The major concern with the paper is that the analysis relies on weak correlations to make strong causal statements about flood event complexity and flood losses. Machine learning models, like a traditional regression model, are subject to the same limitation that correlation does not equal causation. The “event complexity” metric could be a good predictor of flood losses simply because it is correlated with/confounded by any number of other factors that affect flood losses across regions. In other words, without a strong causal inference framework supporting the analysis, there is a good chance that the results are a spurious correlation created by a variety of underlying factors. Some points the authors should consider and control for in any analysis to identify causal influences on reported flood losses:
- Flood reporting is typically incomplete, with many events missing, especially smaller events. What is the effect of missing data on the analysis, especially with temporal or spatial biases in more complete vs. incomplete data?
- Regions with more accurate reporting (include more smaller events), might have lower average flood intensity and lower average flood losses per event. In this case, the average flood losses might be dictated by reporting more so than actual flood risk for a region.
- Compound hazards are increasing in frequency over time – what other temporal patterns in flood losses might have occurred (such as reporting trends and/or changes in exposure/vulnerability) that would make compound hazards correlated with flood losses for spurious reasons?
- How do you account for the fact that losses for some compound events might be reported together (i.e. losses reported for a compound extreme wind-flood event might include both flood and wind damage)? It would be rare for compound event impacts to be separated out by hazard within typically hazard databases.

A much stronger approach would be to apply an event-scale analysis to look at expected losses from compound vs. single flood events, while controlling for confounding influences between locations and over time. Additionally, while the authors include good reasoning why different types of compound events could theoretically lead to higher flood losses, I am not convinced that their new event complexity metric is really sufficient to capture the complexity of these physical processes and exposure/vulnerability interactions.

Line-by-line comments:

Abstract: “our ensemble machine learning models predict that flood losses for compound events are 3.4 times higher than for single events.” This statement is not supported by the analysis. In reality, what the analysis says is that regions with a higher compound event complexity experience average reported losses that are 3.4 times higher than regions that don’t experience compound flood events.

Page 2 paragraph 2: “To effectively capture the diverse interactions of compound hazards in a region, we introduce a novel metric, termed event complexity.” I find the term event complexity to be confusing, because it does not refer to the complexity of individual events. Something like “regional event complexity” would better convey that this is a single metric for each region.

Figure 2: the labels in this figure are too small to read

Page 5 paragraph 2: “A higher value reflects greater diversity, indicating that regions with more varied types of compound events may face greater challenges in preparedness due to unpredictability.” It is not necessarily true that compound events are more unpredictable.

Page 7 paragraph 1: “in low-vulnerability regions, the average SHAP value for complexity is about –0.14, indicating a slight negative effect on losses
compared to the model’s average prediction across the dataset, whereas in high-vulnerability regions, the average SHAP is around 0.2, reflecting a clear positive contribution that amplifies flood damages relative to the baseline” Can you explain why the effect of compound event complexity would be opposite in low-vulnerability vs high-vulnerability regions?

Page 9 paragraph 3: “Missing values for the return period were filled by first replacing them with the mean return period within each NUTS-3 group.” Why would return period values be missing? Would this have the effect of artificially lessening the important of return period as a predictor because noise has been added to the return period values?

Figure 7: this figure shows that there is significant overfitting in the trained regression models, which makes me question the

reliability of the results. The implications of overfitting should be addressed in the text. Additionally, I would suggest the authors try to implement regularization methods to reduce overfitting and check whether the results are the same.

(Remarks on code availability)

Reviewer #3

(Remarks to the Author)
Compound events can substantially exacerbate disaster impacts. This study uses a pan-European flood-impact dataset to examine how compounding hazards relate to flood losses. The authors report that ~80% of recorded floods involve compound hazards, especially among the most damaging 1% of events. They further build up a machine-learning model using a proposed complexity index, exposure, vulnerability, and other covariates. With explainable AI, they argue that higher compound-hazard complexity largely amplifies flood damage.
The perspective and findings are quite interesting and relevant. However, the methodology is too unclear and internally inconsistent to support the conclusions for a high-impact journal like Nature Communications. I therefore recommend reject and encourage resubmission after major methodological clarification and revision.

Major comments:
The paper applies different lead/overlap windows across hazard types (e.g., heat/cold–flood defined on [-14,0]days vs. others using windows up to 56 days). Please justify these choices and test sensitivity to alternative windows.

Drought is defined as a 7-day moving average of discharge below the climatological 5th percentile. This is very short for "drought." Ref. 43 uses a minimum duration ≥10 days. I encourage adopting a longer minimum duration (or providing a sensitivity analysis) to better reflect hydrological drought processes. Also, pairing a short-duration drought with a 56-day compound window likely inflates "drought + wet-sequence" classifications (i.e., many "droughts" will be followed by ordinary wet spells within 56 days). Please reconcile this duration mismatch and quantify its impact.

The paper states: "When more than one compounding hazard was associated with a recorded flood event, the recorded flood event was duplicated…". This has major implications. As Supplementary Fig. 4a suggests, multi-hazard co-occurrence is common. Duplication (multi-counting) inflates sample size, biases relative frequencies, and propagates to most figures. Given strong dependence pairs (e.g., heat and drought, windy and wet conditions), some types will be counted multiple times, altering Fig. 2c and exaggerating contrasts among categories.

Again, duplicating compound events will systematically alter the Shannon entropy, since it changes both the sample size and the proportions of each event type, thereby distorting the results and making them harder to interpret.

"However, windstorm–flood, wet sequences, and drought–flood remain the most frequent archetypes regardless of the window choice." The ranking (windstorm–flood, wet sequence, drought–flood) is influenced not only by cross-dependence but also by base hazard frequency, which itself depends on each hazard's definition, thresholds, and duration rules. Please clarify definitions and show the spatial frequency maps of each associated hazard to avoid misinterpretation.

"A compound event was recorded when the date of an associated hazard (drought, flood, heatwave, coldwave, or windstorm) fell within a defined temporal window relative to the flood start date and both occurred in the same region." — This is unclear. What do the authors mean by "the same region"? A single recorded flood can affect multiple NUTS-3 regions. Is a flood–hazard pair counted as compound if the associated hazard occurs in any one affected NUTS-3 region, or must it occur in all affected regions? If the criterion is satisfied by any single NUTS-3 region, then larger, more widespread floods will be mechanically more likely to be classified as compound event, which could directly drive the pattern in Fig. 2c.

The model's test-set performance appears unsatisfactory: the mean R^2 across the 300 random splits is only ~0.2. This makes the subsequent SHAP-based explanations less convincing. Do the authors compare their performance with results reported in the literature?

Minor comments:
"If no anomaly or hazard is present within the selected window, the flood event is classified as a single event." What exactly is the "selected window"? Since hazard-specific windows have different lengths, do you mean the 56-day window?

"Single flood events (no compounding hazard) are dominant in 33% of regions with at least one recorded flood event (1081/1456)." What do 1081/1456 refer to precisely—regions or events? Please clarify the denominator and the statistic.

"no recorded impact, bottom 50%, 50th–10th percentile, 10th–1st percentile, and the top 1% most damaging event", should be 50th-90th percentile, 90th-99 percentile and top 1% most damaging event.

Figure 6: What is meant by "pre-drought," "pre-flood," etc.? Please define these terms clearly.

Figure 6. The correlation (~0.6) between complexity and pre-drought is quite high. Do the authors have an explanation (e.g., regional/seasonal effects, co-definition, or modeling artifacts)?

(Remarks on code availability)

Version 1:

Reviewer comments:

Reviewer #1

(Remarks to the Author)
Many thanks to the authors for their clear and thorough responses to my comments and concerns. I think that all issues raised in my initial review have been satisfactorily addressed, and I now consider the manuscript suitable for publication in Nature Communications.

(Remarks on code availability)

Reviewer #3

(Remarks to the Author)
Thank the authors for the revision and for addressing most of my previous comments. However, several key issues remain unclear and should be resolved before the manuscript can be considered for publication.

1.The revised three mutually exclusive categories (single-flood, 1 compound hazard, 2+ compound hazards) are an improvement. However, larger floods (affecting more NUTS-3 regions) are mechanically more likely to overlap with additional hazards. Therefore, the reported higher losses in compound categories may primarily reflect a larger flood footprint, rather than a compound amplification effect. To clarify this, please (1) plot the distribution of flood footprint (e.g., number of affected NUTS-3 regions per event) across the three categories; and (2) compare losses between single vs. compound events conditional on similar footprint (e.g., stratification/matching, or regression controlling for footprint and/or event fixed effects) to test whether compound events still increase losses given comparable flood extent.

2.As shown in Figure 3d and stated in the manuscript ("The total effect of complexity … indicating that part of the impact is mediated through return period"), can the authors explain how "complexity" would influence the flood return period itself? Please provide a defensible mechanism to help readers understand this assumption. Alternatively, could return period (or event scale) be a confounder correlated with compound overlap probability, rather than a mediator?

(Remarks on code availability)

Reviewer #4

(Remarks to the Author)
I was asked to look specifically at whether the revisions address the concerns raised by Reviewer 2, because Reviewer 2 was not available to look at this revision. After reading the reviewer's comments alongside the revised manuscript and author responses, I think the main issues have been handled appropriately.

Reviewer 2's main concern was that the original framing made causal claims without a causal framework. The revised manuscript now includes a Double Machine Learning / Causal Forest analysis, and the text has been adjusted to emphasize associations rather than firm causation; the assumptions and limits of what can be inferred from observational data are spelled out more clearly.

The reviewer also raised several questions about data completeness, possible reporting biases, and time-varying changes in exposure or vulnerability. The expanded Methods section now explains the completeness of the HANZE dataset in more detail and draws on previous work to show how missing or underreported events might affect the analysis. The manuscript also clarifies that the temporal analysis applies to changes in hazard frequency, not losses, and includes additional trend information.

On the question of multi-hazard losses being lumped together, the authors now describe how HANZE separates losses by hazard type and point to earlier work where this is documented. This directly responds to the reviewer's concern about confounding between wind-related and flood-related damages.

The line-by-line comments also appear to be addressed. The abstract wording has been corrected; the complexity metric has been renamed to avoid confusion; figure readability has been improved; the discussion around unpredictability and the SHAP patterns has been clarified; the imputation of missing return periods is explained more transparently; and the authors now comment directly on overfitting, regularization, and the stability of their results.

Taken together, I believe the revisions respond adequately to Reviewer 2’s critiques.

(Remarks on code availability)

Version 2:

Reviewer comments:

Reviewer #3

(Remarks to the Author)
Thank you to the authors for the clear and thorough response to my comments. I think that most of the issues I raised have been adequately addressed. I only have one minor comment:

Why is the loss difference between single-flood and compound-flood events large and significant for small floods, but not for large floods? One possible explanation is that, for small floods, the proportion of NUTS3 regions experiencing compound events is relatively high. By contrast, for large-extent floods, the proportion of NUTS3 regions experiencing compound events relative to the total affected flood extent is lower, making it more difficult to distinguish the loss difference.

(Remarks on code availability)

Open Access This Peer Review File is licensed under a Creative Commons Attribution 4.0 International License, which permits use, sharing, adaptation, distribution and reproduction in any medium or format, as long as you give appropriate credit to the original author(s) and the source, provide a link to the Creative Commons license, and indicate if changes were made.
In cases where reviewers are anonymous, credit should be given to 'Anonymous Referee' and the source.
The images or other third party material in this Peer Review File are included in the article’s Creative Commons license, unless indicated otherwise in a credit line to the material. If material is not included in the article’s Creative Commons license and your intended use is not permitted by statutory regulation or exceeds the permitted use, you will need to obtain permission directly from the copyright holder.
To view a copy of this license, visit https://creativecommons.org/licenses/by/4.0/

REVIEWER COMMENTS

Reviewer #1 (Remarks to the Author):

This manuscript compiles and analyses a European-wide dataset of flood impacts (economic losses) associated with compounding hazards. In my view, its most significant contributions are the dataset itself (including the proposed new metric) and the finding that all the costliest events were linked to multiple hazard types. The examination of how vulnerability and exposure influence the relationship between hazard complexity and losses is also valuable, but weaker in execution in its current form. I welcome this initiative and agree that combining existing impact data with hazard, exposure, and vulnerability information is a promising way to maximise the value of available datasets. That said, the manuscript requires substantial revisions before it can be considered for publication in Nature Communications. Below, I outline my major concerns and suggestions for improvement. This study has strong potential, and addressing the points raised would substantially enhance its clarity, methodological soundness, and interpretive depth.

1. Interpretation of main findings
The manuscript should more clearly acknowledge that feature importance from SHAP analysis does not equate to causality. Greater discussion is also needed on the relationship between hazard complexity and losses. Is it the hazard interactions or the vulnerability interactions that exacerbate impacts? While Table 1 offers a theoretical overview, the dataset could potentially be used to empirically identify which interactions tend to amplify economic losses. The claim that unpredictability drives losses (Results, paragraph 4) would benefit from supporting analysis. In essence, does hazard complexity increase impacts for the same flood severity, or does it instead amplify flood severity, indirectly increasing losses? Controlling for flood severity in the analysis could help clarify this, still open, question. And considering that the model only shows moderate performance, how does this affect your results?
We thank the reviewer for this insightful comment. We have clarified in the manuscript that SHAP values represent associations between predictors and modeled losses, not causal relationships (lines 137-139). To disentangle whether hazard complexity amplifies losses via hazard interactions, vulnerability interactions, or increased flood magnitude (expressed as return period), we added a causal analysis using Double Machine Learning with Causal Forest decomposition. This approach estimates both the total effect of hazard complexity and its direct effects when controlling for return period and vulnerability. After adjusting for flood magnitude, the effect decreases slightly, indicating that part of the influence of complexity operates through increased flood magnitude. In contrast, adjusting for vulnerability has little impact on the estimated effect, suggesting that vulnerability does not mediate the causal relationship between hazard complexity and losses (See Fig. 3d; lines 156-166). We also performed a stratified analysis across Return Period quartiles (Supplementary Fig. 16a,b; lines 713-725). High-complexity events consistently have higher losses, independent of flood magnitude, and SHAP contributions confirm a moderate role of vulnerability. Regarding model performance, while predictive accuracy is moderate, SHAP values are aggregated over 300 random train–test splits, which stabilises inferred associations. Model-independent permutation tests further confirm that compound-hazard events have higher losses than single-hazard floods (lines 115-118, 336-344, Fig. 2d). The

causal estimates remain robust under alternative model specifications (i.e. controlling vulnerability and flood magnitude), reflecting the ability of DML to tolerate imperfect nuisance models. We note that economic loss modeling at the NUTS3 level is inherently challenging, and moderate performance is expected, yet our approach represents a methodological advance.

2. Event duplication and definition
Duplicating records when an event is associated with more than one compounding hazard type is problematic, as it may bias results. A more robust solution could, for instance, be to introduce an additional archetype for these events. Relatedly, the definition of an “event” is unclear. Is it a single HANZE record? Could multiple records represent the same physical event across administrative regions, and is this accounted for in the analysis? Given that the model likely assumes independence between observations, correlated records from the same event could bias outcomes. This should at least be acknowledged, if not addressed analytically.
We thank the reviewer for highlighting this important issue. In the revised analysis, each HANZE flood impact entry is now assigned to one of three mutually exclusive categories—single-hazard flood, flood with one additional compounding hazard, or flood with two or more compounding hazards—eliminating any duplication (Fig. 2; Tables 2–4). Regarding the definition of an “event,” each entry represents a unique HANZE flood impact. We acknowledge that a single physical flood can affect multiple NUTS3 regions, producing correlated entries. As noted in Paprotny et al. (2024b), approximately 33% of HANZE riverine flood entries matched to hydrological reanalysis share the same hydrological cause. We now explicitly recognize that such correlations may affect the independence assumption of the machine-learning model and represent a potential source of bias (lines 121–123). While some correlation between observations is unavoidable in real-world data, we believe our revised categorization substantially reduces bias from event duplication.

3. Sample transparency
The manuscript should report the total HANZE sample size, and the number identified as compound events. The figure of 1,955 compound events is given, but without reference to the total number of records or the extent of duplication.
We thank the reviewer for this important comment on transparency. Under the new, mutually exclusive event categorization described above, we have clarified all relevant information on the HANZE dataset, including the total number of records, the number of unique flood events, the number of compound events, and the extent of prior duplicates (lines 74–78; Tables 2–4).

4. HANZE database description
The description of the HANZE database should be more comprehensive, for instance clarifying data sources, included flood types, disaster inclusion criteria, and potential spatiotemporal reporting biases. The rationale for including only riverine and flash floods should be explained, and whether HANZE’s “compound” category of river + coastal is included. The large number of records with “no recorded impacts” also need clarification; do these represent true zero-loss events or missing data?
The HANZE database is described comprehensively in Paprotny et al. (2024a), including sources (full list: https://naturalhazards.eu/list-of-references), inclusion criteria, and known spatiotemporal reporting biases. We have expanded the Methods section (lines 207–215) to

clarify these points. Coverage is generally comprehensive after 1990, though some countries—particularly in Eastern Europe—have lower completeness earlier. For the period 1981–2020 considered here, coverage is largely sufficient; Paprotny et al. (2025) estimate HANZE captures 92% of total flood economic losses in Europe 1950–2020. We do not include compound river/coastal events due to high variability in relative contributions of each hazard. Events with ‘no recorded impacts’ indicate missing economic loss data, but were included if another impact metric (fatalities, persons affected, or inundated area) exceeded the inclusion threshold. Modeled estimates suggest these zero-record events have, on average, three times smaller losses than events with recorded economic loss.

5. Archetype context and hazard representation
The archetypes are well explained, but their brief descriptions might be moved from the Methods to the Introduction for early context. In the Emilia-Romagna example, consider briefly explaining how drought preconditions exacerbated the flood impacts. My main question related to the archetypes is why you do not analyse precipitation as a form of wet sequence?
We thank the reviewer for this helpful comment. The compound hazard descriptions have been moved from the Methods to the Introduction (lines 48–55) for earlier context. For the Emilia–Romagna event, we now briefly explain (lines 35–37) that extreme rainfall followed an extended drought, which degraded soil structure and reduced infiltration, thereby exacerbating flood impacts. Regarding the representation of wet sequences, we chose antecedent high-flow conditions rather than precipitation because discharge integrates catchment wetness—including soil moisture, groundwater, and storage—providing a more physically meaningful indicator of flood susceptibility. As shown by Staudinger et al. (2025), antecedent hydrological conditions often play a larger role than triggering precipitation in extreme floods. Additional discussion is provided in the Methods Section on compound hazard types.

6. Focus and scope
The particular focus on flood impacts should be highlighted earlier in the Introduction. The paper opens with a general discussion on multi-hazards, but I think the transition to focusing only on flood impacts could be written in a more clear and justified manner. And more importantly, regarding impact classes, the rationale for excluding other impact metrics available in HANZE (e.g., fatalities, affected people) needs to be better justified.
We thank the reviewer for this suggestion. We now highlight the focus on flood impacts earlier in the Introduction (lines 32–33), clarifying the transition from the general multi-hazard context to our study’s specific scope. While the main text focuses on economic losses, we also applied our framework to other HANZE impact metrics—fatalities and affected population—in response to the reviewers’ request. These results are reported in the Supplementary Information (Table 6, Figure 17, lines 726-737). These additional analyses show qualitatively consistent patterns, supporting the robustness of our findings

7. Vulnerability and exposure variables
The rationale for linking social vulnerability data to economic losses needs more theoretical grounding. Economic losses often correlate with asset value rather than social vulnerability. You might want to consider choosing only the vulnerability indicators most relevant to economic damages since this is what you focus on. Moreover:

• Which variables do you mean represent infrastructure vulnerability (mentioned in discussion, first paragraph)?
We revised the text to replace “infrastructure vulnerability” with “economic vulnerability” to more accurately reflect the included predictors.

• The manuscript should also acknowledge and discuss potential collinearity among vulnerability and exposure predictors, and how this might affect the results.
Pairwise correlations between all exposure variables and vulnerability indicators are shown in Supplementary Fig. 13. As noted there, certain predictors exhibit substantial collinearity—for example, the RDH vulnerability index is strongly correlated with population (r = 0.74), reflecting the fact that population is one of its input variables. Such collinearity can reduce interpretability of individual model associations, including SHAP value decompositions. However, it does not affect the main conclusions of our study, which rely on the consistent and robust relationship observed between compound hazard complexity and flood losses.

• If kept in analysis, I think that more detail is needed on the vulnerability datasets, including a more thorough reporting of the factors behind the RDH VI index. And is the global SoVI applicable to a European context?
We have expanded the description of the RDH-VI index and its components in the revised Methods (lines 348–354). While GlobE-SoVI was developed for fatality risk and presents global coverage, its underlying dimensions - such as education, inequality, age structure, and institutional capacity - also influence damage severity in the European context. We clarify this in the text (lines 359-364).

• Regarding exposure, choice of built-up area as the sole exposure metric should be explained, why not population data?
We focus on built-up areas rather than population because it better captures the physical assets exposed to floods. Moreover, as shown by Schiavina et al. (2022), built-up areas in Europe are expanding faster than population, making them a more relevant metric for economic exposure.

• Regarding data aggregation, why use average exposure and vulnerability values over the study period when dynamic data are available? And the methodology for spatial aggregation is unclear. While averaging across NUTS regions may be appropriate for some variables, its meaning is less obvious for datasets such as built-up area. How should the resulting averaged values be interpreted?
Our outcome variable is the log of average economic losses per NUTS3 region over 1981–2020, which is static. To ensure consistency, vulnerability and exposure covariates are temporally averaged only where needed (RDH-VI, GDP, HDI, Gini, built-up area, population), while Return Period is averaged across all events affecting each NUTS3 region. SoVI is static by definition. RDH-VI varies slightly year-to-year but shows limited temporal dynamics, as expected for socioeconomic indicators (Sibilia et al., 2024). Compound-hazard variables (preflood, predrought, preheat, precold, prewind) are summed across events to construct the hazard-complexity metric. All datasets are already harmonised at the NUTS3 level; therefore, no spatial averaging is applied. We acknowledge that this approach does not capture short-term dynamics, but it is consistent with our focus on long-term, region-level economic loss patterns.

8. Trends and statistics

When identifying trends, specify whether statistical tests were performed. Clarify whether trends in drought or heat compound events mirror trends in individual hazards or reflect a growing compound nature.

The rationale for using average flood damages (rather than another statistic) should be discussed.

The use of flood return periods should also be more clearly described, including data coverage and calculation method.

We thank the reviewer for these helpful suggestions. We now clarify in the manuscript how trends were identified and assessed (lines 330-337). Specifically, we evaluated the statistical significance of trends in both single-hazard and compound events using the Mann–Kendall test. These results are now reported in Supplementary Table 5 and illustrated in Supplementary Figures 5 and 6. We also added a dedicated subsection discussing the extent to which temporal trends in compound events reflect trends in the individual hazards versus changes in their co-occurrence (lines 636–661). To support this, we provide regional maps of the occurrence of all five considered hazards at the NUTS3 level (Supplementary Figures 8–10). We have also substantially expanded the description of how flood return periods were computed (lines 258-267). These were derived from a hydrological reanalysis for 1950–2020 (Tilloy et al., 2025), which provides daily discharge at 1.8 km resolution across the study domain. Following Mentaschi et al. (2016), we applied transformed-stationary extreme value analysis to remove non-stationarity and fitted a generalized Pareto distribution to peaks-over-threshold to obtain stationary return period estimates at each grid cell. Event-level return periods were then computed as the geometric mean across all contributing river grid cells. We also corrected the reference: the detailed methodology is described in Paprotny et al. (2024), not in the previously cited publication.

9. Terminology and clarity

Across the manuscript, the terms “events”, “archetypes”, and “hazard types” appear to be used somewhat arbitrary and interchangeably. For example, in reference to the “event complexity” metric, described as measuring “how varied the mix of events is”, but the measure is really the mix of hazard types, right? This distinction should be clarified and referred to in a consistent manner throughout the manuscript.

We thank the reviewer for highlighting this important point. We have now clarified and standardized our terminology throughout the manuscript. In the revised text, the term events is used exclusively to denote observed HANZE flood impact records. The term hazard types refers to the six categories considered in the analysis (one single-hazard flood type and five compound flood–hazard combinations). We now use compound hazard types specifically when referring to the five flood–hazard pairs. We agree that the “event complexity” metric captures the diversity of hazard types occurring within a region, rather than variation within individual events. To avoid ambiguity, we replaced the term archetypes with the more precise compound hazard types, and updated the description of the complexity metric accordingly. These changes improve conceptual clarity and ensure consistent use of terminology. All definitions have been revised and consolidated in the Methods section, and the terminology has been harmonized across the manuscript.

More Detailed Comments

• Abstract: The phrase “the Early Warnings for All initiative” may not be familiar to all readers. A brief explanation would be helpful.
Removed.

• Introduction (first sentence): The term “driver” may not be clear to all readers. Consider clarifying.
Changed to “hazard” also for consistency with the terminology of the rest of the manuscript.

• Table 1: The table caption would benefit from explaining what the numerical values represent, rather than repeating the list of archetypes (which are already shown in the table).
Days, specified.

The numbers themselves could instead be explained in the Methods section. Also, the in-text listing of archetypes is not fully consistent with Table 1, consider aligning these for clarity.
• Figure 1: Consider using the colour white to represent “zero” consistently across all panels. Additionally, it might be more informative to also visualize the accumulated frequency of events and economic losses across the administrative regions.
We revised them and also added a figure showing cumulative economic losses at the NUTS3 level in Supplementary Information (Fig. 12a).

• Figure 2: Panel a: The text above the pie charts is too small to read; clarify what one dot represents and define “MH events.” Panel c (should be b): Clarify what the dots with connected lines represent. Panel b (should be c): Clarify how 10-year averages were calculated at the start and end of the analysis period.
10-year averages at the beginning and end of period that years outside of periods have no values, they therefore represent averages of years with values centered on the given year, meaning that the average for 1980 is in fact a 6-years average (1980-1985).
Changed with yearly proportions.

• Results, paragraph 3: The phrase “We group newly created events” is unclear—what is meant by “newly created”? Also, providing information on how the losses (e.g., category intervals) differ between impact classes would be informative.
Changed.

• Results, paragraph 4: Use a single, consistent term for the new metric rather than multiple names.
Done.

• Results, paragraph 5: Specify the modelling approach more clearly, “supervised learning framework” is vague.
Changed into ensemble ML.

• Novel metric: The description of the novel metric should also appear in the Methods section for completeness.
Added, lines 326-329.

• Supplementary Information, Figure 5: Clarify the meaning of “chosen for enhanced visual clarity.”
Changed.

Reviewer #2 (Remarks to the Author):

I have reviewed the paper “Compounding hazards increase flood economic losses across Europe” by Ronco et al. In this paper, the authors analyze an existing database of recorded floods and flood impacts across Europe over 1981-2020. For each recorded flood, the authors analyze atmospheric reanalysis data, hydrologic reanalysis data, and/or observations to determine whether the flood occurred simultaneously or following extreme heat, extreme cold, drought, extreme wind, or wet antecedent conditions. Floods associated with one of these other events are classified as compound events.

To analyze flood impacts associated with compound events, the authors first define a “compound event complexity” for each EU NUTS-3 region, which is the cross-entropy of the number of compound events of each type in that region. The compound event complexity is then used as a predictor in a Gradient Boosting Regression machine learning model to predict average flood losses for each region. Other predictors to the model include average flood intensity and metrics summarizing exposure and vulnerability in each region. Using Shapley values (an explainable AI method to analyze the influence of different predictor variables), the authors claim that their event complexity metric is the most important driver of higher average flood losses in a region. Ultimately, this leads to statements that

While this paper explores an important topic of how floods (including compound flood hazards) affect flood losses, my assessment is that the methodology is not sufficient to support the types of causal claims that the authors make in the text. I have outlined some of these main concerns below. Overall, I would not consider this paper currently suitable for publication.

Main Comments:

The major concern with the paper is that the analysis relies on weak correlations to make strong causal statements about flood event complexity and flood losses. Machine learning models, like a traditional regression model, are subject to the same limitation that correlation does not equal causation. The “event complexity” metric could be a good predictor of flood losses simply because it is correlated with/confounded by any number of other factors that affect flood losses across regions. In other words, without a strong causal inference framework supporting the analysis, there is a good chance that the results are a spurious correlation created by a variety of underlying factors.
We thank the Reviewer for raising this important concern. We fully agree that our SHAP-based results capture associations, not causal relationships, and that correlation alone cannot support strong causal claims. To address this limitation, we have now extended the analysis with an explicit causal-inference framework. Specifically, we implemented a Double Machine Learning (DML) approach combined with a Causal Forest decomposition, treating hazard complexity as the “treatment” and log-transformed flood losses as the

outcome. This procedure is designed to adjust for high-dimensional confounding while providing orthogonalized estimates of treatment effects. As described in the revised manuscript (Figure 3d; lines 156–166 and 403-423), the estimated average treatment effect is positive and statistically significant. This suggests that higher compound-event complexity is associated with increased flood losses even after accounting for socioeconomic, exposure, and hazard-related confounders. We emphasize in the revised text that, as with any observational study, causal interpretation remains conditional on the assumption that all major confounders are observed and properly adjusted for—an assumption that cannot be fully verified. We therefore frame our findings as evidence of a robust and mechanistically plausible association rather than a definitive causal claim.

Some points the authors should consider and control for in any analysis to identify causal influences on reported flood losses:

- Flood reporting is typically incomplete, with many events missing, especially smaller events. What is the effect of missing data on the analysis, especially with temporal or spatial biases in more complete vs. incomplete data?
- Regions with more accurate reporting (include more smaller events), might have lower average flood intensity and lower average flood losses per event. In this case, the average flood losses might be dictated by reporting more so than actual flood risk for a region.

We thank the reviewer for raising these important points on reporting completeness and potential spatial or temporal biases. In Paprotny et al. (2025), using hydrological reanalysis and impact modelling, it was estimated that HANZE flood events with reported economic loss covered 67% of all economic loss from floods in Europe during 1950-2020. HANZE events with missing economic loss constituted an estimated 25%, and flood events not covered by HANZE the remaining 8%. It was also shown that on average, events lacking economic loss data incurred losses three times smaller than those with reported losses. The difference is even greater for events missing entirely from the record, with average losses more than five times smaller. Paprotny et al. (2024a) performed a qualitative analysis of the completeness for all countries, and also compared the database with others, showing a far higher level of completeness and quality of data than other databases, such as e.g. EM-DAT. The coverage is less comprehensive before 1990, but comprehensive during the period analysed in this study. See also expanded Methods lines 207-215.

- Compound hazards are increasing in frequency over time – what other temporal patterns in flood losses might have occurred (such as reporting trends and/or changes in exposure/vulnerability) that would make compound hazards correlated with flood losses for spurious reasons?

We thank the reviewer for raising this point. Our analysis examines temporal trends in hazard occurrence, not in flood losses. In response to this comment and similar ones from Reviewer 1, we revised Figure 2c to show trends in the frequency of single versus compound events (distinguishing single, flood + 1 compound hazard, and flood + 2 or more compound hazards); we do not analyse temporal trends in loss magnitudes, nor do we claim that losses increased over the study period. We quantify hazard-type trends using the Mann–Kendall test (Fig. 2c), and in the Supplementary Information we decompose trends by hazard pair (e.g., drought–flood, heat–flood) and compare them to regional trends of individual hazards (non-compound), as detailed in Section Temporal trends in single and compound hazards (lines 636-661, Table 5, Figures 5 and 6). Regarding potential effects of

exposure and vulnerability changes on losses, recent evidence (Paprotny et al., 2025) shows that decreasing vulnerability and increasing exposure have largely compensated each other, suggesting these trends do not bias our regional-level analysis of compound hazard impacts.

- How do you account for the fact that losses for some compound events might be reported together (i.e. losses reported for a compound extreme wind-flood event might include both flood and wind damage)? It would be rare for compound event impacts to be separated out by hazard within typically hazard databases.

In HANZE, much effort was taken to use local sources to separate those effects, in contrast to e.g. EM-DAT, where multi-hazard events are considered together. Windstorm losses primarily could be bundled together with coastal and compound flooding, which are not analysed here. For riverine events, the biggest issue is separating landslide-related losses. We refer to Paprotny et al. 2024 for all details on this.

A much stronger approach would be to apply an event-scale analysis to look at expected losses from compound vs. single flood events, while controlling for confounding influences between locations and over time. Additionally, while the authors include good reasoning why different types of compound events could theoretically lead to higher flood losses, I am not convinced that their new event complexity metric is really sufficient to capture the complexity of these physical processes and exposure/vulnerability interactions.

We thank the reviewer for this comment. We have included a model-independent analysis at event level by using simple permutation tests, and results are presented in Figure 2d (lines 114-118). The outcomes are consistent with the NUTS3 level analysis. Event-level loss modelling would require a pixel- or building-scale representation of flood magnitude (i.e. return period) and exposure in order to simulate the physical damage processes associated with individual floods. However, the HANZE loss data are reported at the NUTS3 or multi-NUTS3 level, which is too coarse to support such modelling without strong and unverifiable downscaling assumptions. For this reason, our study adopts a regional, risk-oriented approach, analysing how the mix of compound hazard types within each NUTS3 unit relates to historical average flood losses. In this context, the complexity metric is not intended to reproduce detailed physical interactions at event scale, but to provide a simplified, data-driven summary of multi-driver occurrence that supports continental-scale comparison and the development of multi-hazard risk indicators.

Line-by-line comments:

Abstract: “our ensemble machine learning models predict that flood losses for compound events are 3.4 times higher than for single events.” This statement is not supported by the analysis. In reality, what the analysis says is that regions with a higher compound event complexity experience average reported losses that are 3.4 times higher than regions that don’t experience compound flood events.

We agreed and changed the text.

Page 2 paragraph 2: “To effectively capture the diverse interactions of compound hazards in a region, we introduce a novel metric, termed event complexity.” I find the term event complexity to be confusing, because it does not refer to the complexity of individual events.

Something like “regional event complexity” would better convey that this is a single metric for each region.
We agree and update the text: the metric is now introduced as ‘compound hazard complexity’ and subsequently referred to as ‘complexity’ for brevity.

Figure 2: the labels in this figure are too small to read
Increased font size.

Page 5 paragraph 2: “A higher value reflects greater diversity, indicating that regions with more varied types of compound events may face greater challenges in preparedness due to unpredictability.” It is not necessarily true that compound events are more unpredictable.
We agree and revise the text: greater diversity may challenge preparedness, without implying higher unpredictability.

Page 7 paragraph 1: “in low-vulnerability regions, the average SHAP value for complexity is about –0.14, indicating a slight negative effect on losses
compared to the model’s average prediction across the dataset, whereas in high-vulnerability regions, the average SHAP is
around 0.2, reflecting a clear positive contribution that amplifies flood damages relative to the baseline” Can you explain why the effect of compound event complexity would be opposite in low-vulnerability vs high-vulnerability regions?
The apparent opposite effects of hazard complexity in low- versus high-vulnerability regions reflect the fact that SHAP values measure marginal contributions relative to the model’s baseline prediction, not absolute losses. In low-vulnerability regions, resilient systems mitigate the impact of complex hazard interactions, leading to slightly negative SHAP values. In high-vulnerability regions, the same complexity amplifies losses, producing positive SHAP values. We expanded the discussion on the interactions among complexity, vulnerability and flood return period, see Figure 16 (lines 713-725).

Page 9 paragraph 3: “Missing values for the return period were filled by first replacing them with the mean return period within each NUTS-3 group.” Why would return period values be missing? Would this have the effect of artificially lessening the important of return period as a predictor because noise has been added to the return period values?
We agree that imputing with group means may reduce the observed predictive strength of return period, and we now note this as a limitation in the Methods section lines 258-267. Return-period values are derived from hydrological reanalyses and simulations (Paprotny et al., 2025), and some observed floods have no matching modelled discharge anomaly at the relevant catchment scale. In these cases, missing values were imputed using the mean return period within the same NUTS3 region and, if still missing, within the same event ID. Because this approach reduces within-group variability, it may lead to a conservative (i.e., underestimated) estimate of the predictive importance of the return period.

Figure 7: this figure shows that there is significant overfitting in the trained regression models, which makes me question the reliability of the results. The implications of overfitting should be addressed in the text. Additionally, I would suggest the authors try to implement regularization methods to reduce overfitting and check whether the results are the same.
We thank the reviewer for raising this important point. We agree that overfitting must be carefully evaluated in supervised models applied to impact data. In our case, several

elements of the modelling pipeline already act as regularization mechanisms. The hyperparameter search constrains the model to shallow trees (maximum depth 2–4), relatively high `min_samples_split` and `min_samples_leaf`, and subsampling, all of which strongly penalize overly complex trees. These constraints represent standard forms of structural regularization in gradient boosting. Consistent with this, the model shows limited overfitting: while it explains on average 47% of the variance in the training data and 22% in the test data, the mean train/test MSE ratio is 0.73, indicating that performance does not degrade sharply on held-out samples. Importantly, our interpretation framework—SHAP values aggregated over 300 random train/test splits—produces stable and well-converged estimates of feature contributions and interactions across repetitions. We now discuss these aspects and remaining limitations more explicitly in Supplementary Information (lines 693-712).

Reviewer #3 (Remarks to the Author):

Compound events can substantially exacerbate disaster impacts. This study uses a pan-European flood-impact dataset to examine how compounding hazards relate to flood losses. The authors report that ~80% of recorded floods involve compound hazards, especially among the most damaging 1% of events. They further build up a machine-learning model using a proposed complexity index, exposure, vulnerability, and other covariates. With explainable AI, they argue that higher compound-hazard complexity largely amplifies flood damage.
The perspective and findings are quite interesting and relevant. However, the methodology is too unclear and internally inconsistent to support the conclusions for a high-impact journal like Nature Communications. I therefore recommend reject and encourage resubmission after major methodological clarification and revision.

Major comments:

The paper applies different lead/overlap windows across hazard types (e.g., heat/cold–flood defined on [-14,0]days vs. others using windows up to 56 days). Please justify these choices and test sensitivity to alternative windows.
We thank the reviewer for pointing that out. The choice of overlap windows is indeed a critical aspect of the analysis. We considered three temporal windows: short (14 days), medium (28 days), and long (56 days) and discussed the sensitivity of our results to the usage of these 3 windows (Methods and SI Figure 4b). We justify the choices of our windows by prior knowledge taken from the literature on hazard and vulnerability interrelations between flood and compounding hazards. We have also strengthened this justification in the revised manuscript, see Methods section on compound hazards.

Drought is defined as a 7-day moving average of discharge below the climatological 5th percentile. This is very short for “drought.” Ref. 43 uses a minimum duration ≥10 days. I encourage adopting a longer minimum duration (or providing a sensitivity analysis) to better reflect hydrological drought processes. Also, pairing a short-duration drought with a 56-day compound window likely inflates “drought + wet-sequence” classifications (i.e., many “droughts” will be followed by ordinary wet spells within 56 days). Please reconcile this duration mismatch and quantify its impact.

We thank the reviewer for this valuable comment. To better represent hydrological drought processes and ensure consistency with the drought literature and with the 56-day compound-event window, we refined our drought identification procedure. Specifically, we now apply a pooling strategy following Fleig et al. (2006), merging consecutive low-flow episodes separated by <30 days into a single drought event. This substantially reduces fragmented short-duration episodes and yields more coherent hydrological drought clusters. We additionally performed a sensitivity analysis using minimum drought durations of ≥0 days (no minimum), ≥7, ≥10, and ≥14 days (see SI subsection Sensitivity of drought–flood compound events to minimum drought duration, lines 662-678). For each threshold, we (i) quantified changes in the number of compound drought–flood events relative to single-flood events (Supplementary Fig. 7a) and (ii) compared the corresponding loss distributions. Kolmogorov–Smirnov tests on log-transformed flood losses produced p-values close to 1 across all thresholds and event types, indicating statistically indistinguishable loss distributions (Supplementary Fig. 7b). Based on these results—and to align our event definition with both Ref. 43 and the 56-day compound window—we now adopt a minimum drought duration of 10 days in the main analysis.

The paper states: "When more than one compounding hazard was associated with a recorded flood event, the recorded flood event was duplicated…". This has major implications. As Supplementary Fig. 4a suggests, multi-hazard co-occurrence is common. Duplication (multi-counting) inflates sample size, biases relative frequencies, and propagates to most figures. Given strong dependence pairs (e.g., heat and drought, windy and wet conditions), some types will be counted multiple times, altering Fig. 2c and exaggerating contrasts among categories.

Again, duplicating compound events will systematically alter the Shannon entropy, since it changes both the sample size and the proportions of each event type, thereby distorting the results and making them harder to interpret.

We thank the reviewer for raising this important point. In our revised analysis, each flood event is now classified into three mutually exclusive categories—single-flood, 1-compound, and 2+ compound hazards—so each event is counted only once (see Figure 2, Tables 2, 3, 4). See also our reply to Reviewer 1. At the regional (NUTS3) level, a flood affecting multiple regions is counted in each region, with losses distributed proportionally, and compound hazards are matched per NUTS. This follows established approaches for compound events (Refs. Jager et al. 2025; Sutanto et al. 2020, Tilloy et al. 2022; Claassen et al. 2023) and avoids biases in relative frequencies or loss estimates. Hazard complexity, measured via Shannon entropy, depends on the distribution of event types rather than absolute counts. A NUTS3 region can experience many floods, but if all belong to the same category, entropy remains low. This allows us to meaningfully link the diversity of compounding hazards with average losses, rather than conflating frequency with complexity.

"However, windstorm–flood, wet sequences, and drought–flood remain the most frequent archetypes regardless of the window choice." The ranking (windstorm–flood, wet sequence, drought–flood) is influenced not only by cross-dependence but also by base hazard frequency, which itself depends on each hazard's definition, thresholds, and duration rules. Please clarify definitions and show the spatial frequency maps of each associated hazard to avoid misinterpretation.

We thank the reviewer for this comment. To clarify the factors influencing the ranking of compound event types, we have now included statistical tests for trends of each single

hazard compared against compound events. This can be found in Supplementary Figures 5 and 6. In addition, we included spatial NUTS3 level maps of occurrences of all the 5 hazards in Figures 8, 9, 10. These additions ensure that the observed compound-event rankings are interpreted in the context of both cross-hazard dependence and the underlying occurrence patterns of each hazard type. See the Supplementary Information section lines 636-661 and Table 5.

"A compound event was recorded when the date of an associated hazard (drought, flood, heatwave, coldwave, or windstorm) fell within a defined temporal window relative to the flood start date and both occurred in the same region." — This is unclear. What do the authors mean by "the same region"? A single recorded flood can affect multiple NUTS-3 regions. Is a flood–hazard pair counted as compound if the associated hazard occurs in any one affected NUTS-3 region, or must it occur in all affected regions? If the criterion is satisfied by any single NUTS-3 region, then larger, more widespread floods will be mechanically more likely to be classified as compound event, which could directly drive the pattern in Fig. 2c.
We thank the reviewer for highlighting this point. The initial matching of floods with other hazards is performed at the NUTS-3 administrative region level. For a HANZE flood record to be considered a compound event, it is sufficient that at least one of the NUTS-3 regions affected by the flood experiences a compounding hazard within the defined temporal window. Each affected NUTS-3 region is compared separately with the anomalies present in that region. After matching, the results are aggregated back to the event level, so that each HANZE flood record represents an independent event. When we refer to "the same region," we mean the same NUTS-3 region. A flood affecting multiple NUTS-3 regions (e.g., NUTS A and NUTS B) is assessed against hazards in A and B separately. As a result, larger floods spanning more regions are naturally more likely to be classified as compound, simply because there are more opportunities for overlap with other hazards. We acknowledge that this could influence the probability of a flood being identified as compound, but this is not an artifact of the method. Rather, it reflects a true feature of compound events: by definition, they occur simultaneously over a region, so more spatially extensive floods inherently have a higher likelihood of coinciding with additional hazards. We have clarified this in the Methods section.

The model's test-set performance appears unsatisfactory: the mean R^2 across the 300 random splits is only ~0.2. This makes the subsequent SHAP-based explanations less convincing. Do the authors compare their performance with results reported in the literature?
We acknowledge in the manuscript the moderate model performance. Nonetheless, the results are based on robust evaluation of SHAP values over several random splitting of the NUTS3 samples. Moreover, in the revised manuscript, we added model-independent permutation tests to show that losses for compound events are significantly higher than those of single floods. Finally, the average treatment effect of the complexity further confirms the main conclusions of the analysis (lines 156-166). In summary, three (largely independent) methods provide evidence towards the same conclusion. As far as we know, there are no existing studies on subnational risk models for losses, highlighting the importance of our analysis and its contribution to the field.

Minor comments:

“If no anomaly or hazard is present within the selected window, the flood event is classified as a single event.” What exactly is the “selected window”? Since hazard-specific windows have different lengths, do you mean the 56-day window?

Yes, the “selected window” refers to the hazard-specific time windows we defined for each compound hazard pair. For each flood event, we check within the corresponding window for the presence of the co-occurring hazard. If none of the conditions for any compound pair is satisfied—that is, no relevant anomaly occurs within the respective window—the flood event is classified as a single event. We clarify this in the revised manuscript.

“Single flood events (no compounding hazard) are dominant in 33% of regions with at least one recorded flood event (1081/1456).” What do 1081/1456 refer to precisely—regions or events? Please clarify the denominator and the statistic.

Clarified in the manuscript lines 74-78, following the revised categorisation into non-overlapping compound event categories.

“no recorded impact, bottom 50%, 50th–10th percentile, 10th–1st percentile, and the top 1% most damaging event”, should be 50th-90th percentile, 90th-99 percentile and top 1% most damaging event.

We refer to 50% lower losses, losses in the 50 to 10 % of most damaging events, the 10%, and then the most damaging 1% of events. We hope this clarifies the chosen scale.

Figure 6: What is meant by “pre-drought,” “pre-flood,” etc.? Please define these terms clearly.

Thanks for the comment. They refer to the total occurrence of e.g. compound droughts in a given NUTS3 region. We clarified it in Supplementary Information.

Figure 6. The correlation (~0.6) between complexity and pre-drought is quite high. Do the authors have an explanation (e.g., regional/seasonal effects, co-definition, or modeling artifacts)?

Thanks for this observation. The actual correlation is 0.55 so moderate. We can interpret it with the fact that droughts often appear together with heatwaves, and thus leading to a higher mix of hazards at NUTS3 level.

Reviewer #3 (Remarks to the Author):

Thank the authors for the revision and for addressing most of my previous comments. However, several key issues remain unclear and should be resolved before the manuscript can be considered for publication.

We thank Reviewer #3 for their careful evaluation of the revised manuscript and for acknowledging that most previous concerns were addressed. Below, we respond to the remaining points by providing additional analyses, plots, and clarifications, following the reviewer's suggestions.

1.The revised three mutually exclusive categories (single-flood, 1 compound hazard, 2+ compound hazards) are an improvement. However, larger floods (affecting more NUTS-3 regions) are mechanically more likely to overlap with additional hazards. Therefore, the reported higher losses in compound categories may primarily reflect a larger flood footprint, rather than a compound amplification effect. To clarify this, please (1) plot the distribution of flood footprint (e.g., number of affected NUTS-3 regions per event) across the three categories; and (2) compare losses between single vs. compound events conditional on similar footprint (e.g., stratification/matching, or regression controlling for footprint and/or event fixed effects) to test whether compound events still increase losses given comparable flood extent.

We thank the reviewer for this important comment. As noted, larger floods naturally affect more NUTS-3 regions and are therefore more likely to be classified as compound events, as larger floods typically have greater spatial extent and longer duration, increasing the likelihood of coinciding with additional hazards. At the same time, larger floods tend to generate higher total losses which could be the result of the large flood footprint and/or the compound effect.We agree that it is important to try to disentangle whether the higher losses observed in compound categories arise solely from larger flood footprints or whether compound conditions amplify losses beyond what would be expected from footprint alone.

To address this concern, we added Fig. 5, showing (i) the distribution of flood footprint (number of affected NUTS-3 regions) across the three categories, (ii) losses stratified by flood footprint into small floods (1–2 NUTS-3) and large floods (≥3 NUTS-3), and (iii) losses normalized by the number of affected NUTS-3 regions. Compound events tend to coincide with larger floods (Fig. 5a). When stratifying and normalizing by footprint (Fig. 5b,c), differences in losses between single and compound events persist, particularly for smaller floods. When stratifying losses by flood footprint, compound events affecting 1–2 NUTS-3 regions exhibit higher log-losses than single-flood events (Fig. 5b). For floods affecting ≥3 NUTS-3 regions, differences between categories are smaller and less clearly separated (Fig. 5b). To

quantify these differences, we applied a random permutation test. For small floods (1–2 NUTS-3), compound events show significantly higher losses than single-flood events ($p = 0.048$). This pattern is consistent with compound events being associated with higher losses among spatially limited floods, beyond differences attributable to footprint alone. Differences are not statistically significant for larger floods ($p = 0.63$), suggesting that for widespread events we do not detect a clear distinction between single and compound classifications. We note, however, that the number of single-flood events with large footprints is limited ($N = 26$), which may constrain statistical power. Indeed, large-scale floods are increasingly likely to manifest as compound hazards, making isolated large floods rare. See lines 644-651 in Supplementary Information.

To further explore the relationship between flood extent, losses, and compound hazards, we analyzed losses normalized by the number of affected NUTS-3 regions per event (Fig. 5c). Median losses per NUTS-3 region are higher for compound events, particularly for events involving two or more additional hazards (median differences: 0.33 for one compound hazard; 0.48 for two or more), with statistical significance for the latter ($p = 0.033$). These results are consistent with compound events being associated with higher impacts beyond differences in flood extent alone. We also stress that our main analysis at the NUTS-3 level using Ensemble Machine Learning and Double Machine Learning directly links compound complexity to losses per-region, providing additional confirmation of this effect. We have clarified the potential coupling between flood size and compound classification in the main text (lines 118–122) and expanded the Supplementary Information (lines 644–651) to describe the additional stratified and normalized analyses.

2.As shown in Figure 3d and stated in the manuscript (“The total effect of complexity … indicating that part of the impact is mediated through return period”), can the authors explain how “complexity” would influence the flood return period itself? Please provide a defensible mechanism to help readers understand this assumption. Alternatively, could return period (or event scale) be a confounder correlated with compound overlap probability, rather than a mediator?

We thank the reviewer for this important question. We agree that the original phrasing could be interpreted as implying a mediation pathway from complexity to return period, and we acknowledge this was overstated. Conceptually, we do not expect a strong mechanism linking regional hazard complexity to flood return period in our framework. In our study, complexity captures the entropy of compound hazard types present in a region — it is a measure of hazard diversity rather than of event intensity or frequency. While specific compound hazard interactions (e.g., wet sequences increasing antecedent soil moisture, or the concurrence of storm surge and riverine flooding) can physically amplify flood severity and thereby influence return periods, these dynamics are embedded in individual hazard interrelations

rather than in regional complexity. We therefore consider it more plausible that return period and complexity act as largely independent primary drivers of losses, rather than one mediating the other.

To move beyond conceptual reasoning and formally assess this structure, we conducted additional Double Machine Learning (DML) analyses estimating the causal effect in both directions. The causal effect of complexity on return period is small (4.2% of the observed return-period range), as is the reverse effect of return period on complexity (5.7% of the observed complexity range). Both are substantially smaller than the direct causal effect of complexity on economic losses (~17% of the loss range). Importantly, controlling for return period in the main DML model reduces the complexity–loss effect only slightly (from ATE = 3.589 to ATE = 2.950), further indicating that differences in flood magnitude explain only a limited portion of the observed association between complexity and losses. Taken together, these results suggest that complexity and return period are largely independent of one another, and that the mediation pathway implied by the original analysis was overstated rather than reflecting a robust causal structure. We have revised the manuscript accordingly (lines 164–172).

REVIEWERS' COMMENTS

Reviewer #3 (Remarks to the Author):

Thank you to the authors for the clear and thorough response to my comments. I think that most of the issues I raised have been adequately addressed. I only have one minor comment:

Why is the loss difference between single-flood and compound-flood events large and significant for small floods, but not for large floods? One possible explanation is that, for small floods, the proportion of NUTS3 regions experiencing compound events is relatively high. By contrast, for large-extent floods, the proportion of NUTS3 regions experiencing compound events relative to the total affected flood extent is lower, making it more difficult to distinguish the loss difference.

We thank the reviewer for this insightful comment. We agree that the 'concentration' of compound drivers within the total flood footprint is a highly plausible explanation for the observed results. As our classification is event-based, a compound designation for a localized flood (1–2 NUTS-3) implies that the compound drivers likely coincide with the bulk of the affected area, resulting in a high 'impact density.' In larger floods, the compound signal may indeed be spatially diluted across a wider footprint where only a subset of regions experience overlapping hazards. We changed the main text lines 174-177 into: “We find that compound effects are most pronounced for localized floods—where overlapping hazards characterize a larger share of the affected area—whereas in very large floods, the signal may be spatially diluted and the total impact increasingly dominated by footprint”.

This interpretation is directly supported by our results in Supplementary Figure 2: when we normalize losses by the number of affected regions (Loss/NUTS-3), the compound signal remains significant. This suggests that the amplification effect is present, but for larger events, it is more clearly captured through 'impact density' than through total aggregate losses. We have added this sentence to the Supplementary Information to acknowledge this spatial scaling logic: “This suggests a dilution effect at larger scales: localized floods are more likely to be compound across their entire footprint, while larger floods may be dominated by regions experiencing only single-hazard conditions.”.
